# Discovery of Unannotated Small Open Reading Frames in Streptococcus pneumoniae D39 Involved in Quorum Sensing and Virulence Using Ribosome Profiling

**DOI:** 10.1128/mbio.01247-22

**Published:** 2022-07-19

**Authors:** Irina Laczkovich, Kyle Mangano, Xinhao Shao, Adam J. Hockenberry, Yu Gao, Alexander Mankin, Nora Vázquez-Laslop, Michael J. Federle

**Affiliations:** a Department of Microbiology and Immunology, University of Illinois at Chicago, Chicago, Illinois, USA; b Department of Pharmaceutical Sciences, University of Illinois at Chicago, Chicago, Illinois, USA; c Center for Biomolecular Sciences, University of Illinois at Chicago, Chicago, Illinois, USA; d Department of Integrative Biology, University of Texas at Austin, Austin, Texas, USA; Carnegie Mellon University

**Keywords:** ribosome profiling, *Streptococcus pneumoniae* D39, small proteins, small open reading frames, virulence, quorum sensing, translation inhibitors, translational control

## Abstract

Streptococcus pneumoniae, an opportunistic human pathogen, is the leading cause of community-acquired pneumonia and an agent of otitis media, septicemia, and meningitis. Although genomic and transcriptomic studies of S. pneumoniae have provided detailed perspectives on gene content and expression programs, they have lacked information pertaining to the translational landscape, particularly at a resolution that identifies commonly overlooked small open reading frames (sORFs), whose importance is increasingly realized in metabolism, regulation, and virulence. To identify protein-coding sORFs in S. pneumoniae, antibiotic-enhanced ribosome profiling was conducted. Using translation inhibitors, 114 novel sORFs were detected, and the expression of a subset of them was experimentally validated. Two loci associated with virulence and quorum sensing were examined in deeper detail. One such sORF, *rio3*, overlaps with the noncoding RNA *srf-02* that was previously implicated in pathogenesis. Targeted mutagenesis parsing *rio3* from *srf-02* revealed that *rio3* is responsible for the fitness defect seen in a murine nasopharyngeal colonization model. Additionally, two novel sORFs located adjacent to the quorum sensing receptor *rgg1518* were found to impact regulatory activity. Our findings emphasize the importance of sORFs present in the genomes of pathogenic bacteria and underscore the utility of ribosome profiling for identifying the bacterial translatome.

## INTRODUCTION

Streptococcus pneumoniae, a major human pathogen, uses signaling mechanisms and gene regulation to alter global gene expression in response to dynamic environments during infection and colonization. Advanced transcriptomic technologies have permitted the identification of novel short RNA molecules in the highly studied model strain D39 of S. pneumoniae, some of which have been implicated in virulence (1–3). However, there is a distinct lack of information about small proteins encoded in the S. pneumoniae genome.

While conventional computational and experimental approaches have been well optimized for the prediction of protein-coding sequences in bacterial genomes, the identification and characterization of small open reading frames (sORFs) encoding proteins of less than 50 amino acids have been limited due to constraints in methodology and analysis. Computational algorithms used to annotate genomes require distinct size cutoffs to prevent an excess of predicted ORFs, leading to a trade-off between strict criteria that limit discovery and weaker stringencies that produce many false-positive predictions (4–6). Furthermore, the intrinsic properties of small proteins, such as their low molecular weight, insufficient ionic charge, low abundance, or poor stability, complicate their isolation and characterization using standard biochemical methodologies (7, 8).

Despite the difficulty in their identification, some small proteins or “microproteins” have been identified in a wide range of organisms and shown to impact metal homeostasis, virulence, cell development, metabolism, intracellular signaling, and other important physiological properties (9–11). For instance, in Bacillus subtilis, the small protein SpoVM (26 amino acids) is involved in spore coat and cortex development, and the deletion of *spoVM* results in a significant decrease in the sporulation efficiency (12). In Staphylococcus aureus, the small RNA (sRNA) RNA III encodes the 26-amino-acid cytotoxic peptide delta-hemolysin (*hld*) whose activity targets host cell membranes for lysis (13). In Escherichia coli, the 42-amino-acid protein MntS regulates intracellular manganese homeostasis, and the 49-amino-acid protein AcrZ enhances resistance to antibiotics through its interaction with the AcrAB-TolC efflux complex (14–16).

Quorum sensing (QS), a mode of bacterial cell-to-cell communication, operates through the production and sensing of low-molecular-weight molecules (pheromones) as intercellular signals for the purpose of coordinating activities among community members (17). Gram-positive bacteria employ peptides as pheromones that are secreted to the extracellular space and subsequently detected by neighboring bacteria. Early studies of natural transformation in S. pneumoniae led to the first discovery of intercellular signaling in bacteria, whereupon the competence-stimulating peptide (CSP) pheromone stimulates a histidine kinase in the development of the competent state for DNA uptake (18, 19). More recently, QS systems of the RRNPP (Rap, Rgg, NprR, PlcR, and PrgX) family, which are widespread among *Firmicutes* (17), have also been identified in S. pneumoniae as determined by genomic evaluation, with as many as eight paralogous systems being present. The RRNPP receptor proteins reside in the cytoplasm; therefore, precursors of the QS peptides are secreted, and the accumulated extracellular peptide ligands must then be reimported into the cell for direct interaction with cognate receptors to control gene expression (20–23). While phenotypes associated with the inactivation of these systems are starting to emerge, their roles in gene regulation remain largely unknown (20, 23, 24). A considerable barrier to identifying and characterizing these and other quorum sensing networks is the lack of appropriate techniques for detecting sORFs encoding signaling peptides (17). There are approximately 42,000 hypothetical sORFs in the S. pneumoniae D39 genome that encode peptides 8 to 50 amino acids in length. Which, if any, of these putative ORFs encode QS pheromones is unknown (17).

Although extensive analyses of the S. pneumoniae transcriptome and random transposon mutagenesis have resulted in the identification of several novel genes and noncoding RNAs (ncRNAs), a thorough understanding of the S. pneumoniae translatome is still missing (3, 25). To identify short protein-coding sORFs, some of which might encode uncharacterized peptide QS pheromones, virulence-related proteins, or other physiologically important microproteins, we conducted antibiotic-assisted ribosome profiling (Ribo-seq). Conventional Ribo-seq identifies actively translated ORFs by deep sequencing ribosome-protected mRNA fragments, providing a global view of all the genomic sites undergoing active translation (26–28). A specialized version of ribosome profiling exploits the ability of pleuromutilin antibiotics (e.g., retapamulin [Ret]) to specifically arrest ribosomes at initiation codons enhancing the signal-to-noise readout at a gene’s start, thus facilitating the detection of protein-coding genes, including sORFs (11, 29). Using Ribo-seq and retapamulin-enhanced Ribo-seq (Ribo-RET) analyses, we identified 114 novel sORFs in the genome of S. pneumoniae D39 and validated translation for a subset thereof. Among these, we identified two sORFs encoding short peptides involved in QS signaling and sORFs within nine previously annotated sRNAs, at least one of which contributes to the fitness and virulence of S. pneumoniae D39.

## RESULTS

### Retapamulin and lefamulin stall the ribosome during translation initiation in S. pneumoniae D39.

A recent study used the pleuromutilin antibiotic retapamulin in combination with ribosome profiling (Ribo-RET) to identify the start codons of protein-coding genes in the E. coli genome and discovered 41 novel sORFs in E. coli (11). Retapamulin, a protein synthesis inhibitor, binds to the 50S subunit during ribosome assembly and traps the ribosome during translation initiation, providing an increased signal-to-noise ratio at translation start sites.

We applied Ribo-RET to identify translated sORFs in the S. pneumoniae D39 genome. A D39 capsule mutant was used for all Ribo-seq experiments, as the presence of capsule complicated the rapid isolation of cells and, hence, ribosomes by filtration or centrifugation for downstream processing. Prior to cell lysis, mid-exponential-phase cultures were treated with 62.5 ng mL^−1^ retapamulin for 2.5 min, which corresponds to 100 times the MIC of the drug (see Fig. S1A in the supplemental material). The metabolic labeling experiments established that a 2.5-min treatment with retapamulin is sufficient to completely stop translation in the cell (Fig. S1B). The polysomes were isolated from the cells using the procedures described in Materials and Methods. However, we found that S. pneumoniae ribosomes tended to dissociate into subunits under conventional conditions of sucrose gradient centrifugation. To preserve ribosome integrity, we increased the concentration of MgCl_2_ in the lysis buffer from 10 mM to 50 mM. This adjustment significantly stabilized the ribosomes and slightly diminished the activity of micrococcal nuclease (MNase) used for the preparation of the ribosome footprints (Fig. S1C). This resulted in a less-precise trimming of the footprints to the ribosome edge and a broader distribution of the footprint lengths. Notably, even after digestion with MNase, all sucrose gradient centrifugation profiles showed the presence of an additional peak whose sedimentation properties are consistent with either underdigested disomes or, as observed in S. aureus, hibernating ribosome pairs (Fig. S1D, E, G, and H) (30). Despite these potential complications, monomeric 70S:mRNA footprint complexes were isolated, and the Ribo-seq library was prepared for Illumina deep sequencing.

10.1128/mbio.01247-22.1 FIG S1 Retapamulin and lefamulin are effective at arresting translation in S. pneumoniae D39. (A) MICs of retapamulin and lefamulin determined in CDM. (B) Assessment of protein synthesis in D39 Δ*cps* treated with 100-fold the MIC of either retapamulin or lefamulin by measuring the incorporation of [^35^S]methionine at specific time points. (C to H) Monosomes were isolated by sucrose gradient fractionation. Bacterial cultures were either untreated (C and F) or treated with 100-fold the MIC of either retapamulin (D and G) or lefamulin (E and H) for 2.5 min. Lysates were digested (F to H) or not (C to E) with 450 U MNase and loaded onto 10 to 40% sucrose gradients. Download FIG S1, TIF file, 2.9 MB. Copyright © 2022 Laczkovich et al. 2022 Laczkovich et al. https://creativecommons.org/licenses/by/4.0/ This content is distributed under the terms of the Creative Commons Attribution 4.0 International license.

10.1128/mbio.01247-22.2 FIG S2 Metagene analysis for all read lengths. (A) Metagene analysis comparing all read lengths positioned relative to the start codon with an offset of 15 nucleotides from untreated and retapamulin- and lefamulin-treated cells. (B) Metagene analysis of all read lengths. Download FIG S2, TIF file, 2.4 MB. Copyright © 2022 Laczkovich et al. 2022 Laczkovich et al. https://creativecommons.org/licenses/by/4.0/ This content is distributed under the terms of the Creative Commons Attribution 4.0 International license.

**FIG 1:**
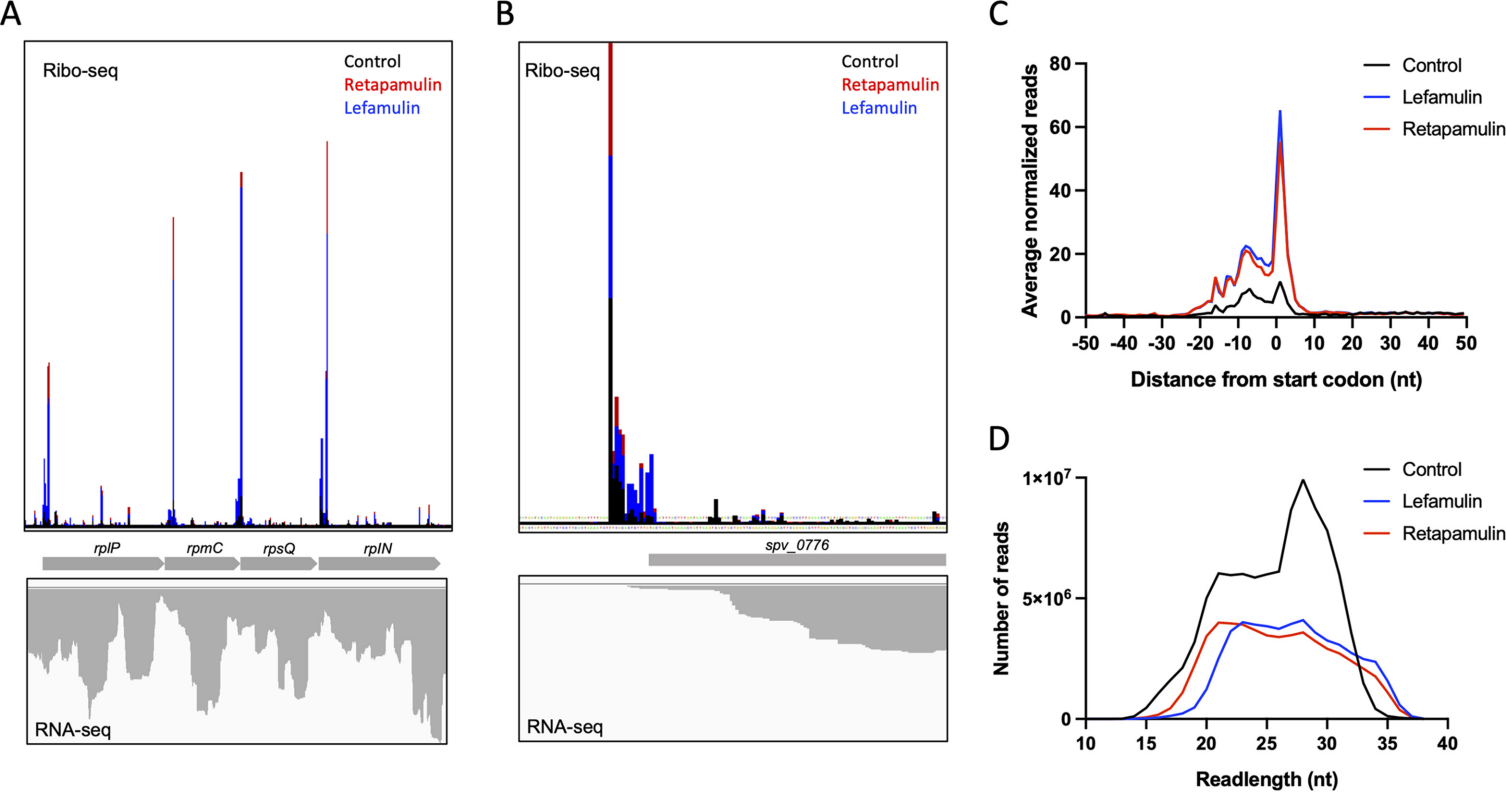
Retapamulin and lefamulin trap the ribosome near the start codon. (A) Ribosome footprint density of S. pneumoniae treated with and without retapamulin and lefamulin. (B) Example of a ribosome footprint stalled prior to the annotated start codon for *spv_0776*. (C) Metagene analysis of ribosome density reads (27 to 35 nt) distributed relative to the annotated start codon. (D) Distribution of the ribosome footprint length.

Ribo-seq data sets from untreated cultures revealed the translation of many annotated S. pneumoniae genes as well as the presence of ribosome footprints in some intergenic regions (Fig. 1A). As predicted, treatment of cells with retapamulin led to the accumulation of ribosomes at the start codons of genes (Fig. 1A). Metagene analysis showed that many ribosome footprints mapped to annotated start codons (Fig. S2B); however, a sizeable fraction of reads placed the ribosome as far as 20 nucleotides (nt) upstream from the annotated translation start sites (Fig. 1B and C). We observed in the raw sequencing data that the distribution of ribosome footprint read lengths ranged from 15 to 35 nucleotides, a surprise considering that studies performed on E. coli typically produce lengths of 28 nucleotides (Fig. 1D) (31). To determine whether the footprint size correlated with the ribosome’s location, read lengths were compared to start codon positioning, and it was found that the reads (27 to 35 nt) aligned best with annotated gene start sites (Fig. S2A). When only reads in this size range were used for mapping, two-thirds of the reads were situated at annotated start codons (Fig. 1C). Finally, to test the possibility that ribosome positioning was dependent on the initiation inhibitor used, we repeated the profiling experiment using lefamulin (Ribo-LEF), another pleuromutilin antibiotic reported to bind tightly to the ribosomal peptidyl transferase center (PTC) (32). The sequencing data sets generated from retapamulin- and lefamulin-treated samples produced outcomes that were nearly identical, reinforcing our confidence in the results of our ribosome profiling (Fig. S1E and H and Fig. S7).

10.1128/mbio.01247-22.7 FIG S7 The ribosome consistently stalls in the 5′ UTR between different sample preparations and antibiotic treatments. Shown is a snapshot of the ribosome footprint density for the *araT* gene. Each picture represents a biological replicate, which was either untreated or treated with retapamulin or lefamulin. Download FIG S7, TIF file, 2.1 MB. Copyright © 2022 Laczkovich et al. 2022 Laczkovich et al. https://creativecommons.org/licenses/by/4.0/ This content is distributed under the terms of the Creative Commons Attribution 4.0 International license.

Similar to Ribo-RET results obtained with E. coli (29), we also identified peaks of ribosomal footprints at putative internal start sites located within annotated ORFs. Such peaks may indicate instances of alternative initiation of translation or nested ORFs, as observed previously in E. coli (29) (Fig. S3).

10.1128/mbio.01247-22.3 FIG S3 Ribosome footprints are present within genes. Examples of Ribo-seq profiles in Streptococcus pneumoniae indicate alternative translation start sites within genes. The gray arrows represent previously annotated genes, and the green arrows indicate alternative open reading frames within annotated genes. Download FIG S3, TIF file, 2.8 MB. Copyright © 2022 Laczkovich et al. 2022 Laczkovich et al. https://creativecommons.org/licenses/by/4.0/ This content is distributed under the terms of the Creative Commons Attribution 4.0 International license.

Overall, the ribosome profiling results indicated that 85% of the annotated S. pneumoniae genome is actively translated under laboratory conditions. As such, antibiotic-assisted ribosome profiling provides a powerful tool to identify the S. pneumoniae translatome.

### Ribo-RET identifies unannotated sORFs in S. pneumoniae D39.

The S. pneumoniae D39 genome encodes ~2,700 intergenic coding sequences with the potential to encode small proteins 10 to 50 amino acids long. To identify true protein-coding regions, three independent sequencing data sets, two utilizing Ribo-RET and one utilizing Ribo-LEF, were used to map the translation initiation sites. Using a computational approach that mapped and quantified ribosome footprints, normalized to genome-wide sequence reads, we used the following criteria to define putative translation start sites of sORF candidates: at least 1 sequence read per million (rpm) mapped within 10 nucleotides of a theoretical sORF start codon (AUG, GUG, CUG, or UUG), and the respective full-length sORF did not overlap an annotated gene. By these criteria, we identified 117 (RET) and 103 (LEF) sORF candidates. In some instances of neighboring putative start codons, manual assessment of the coding region resulted in a refined list of 114 novel sORF candidates, designated *rio* (Ribo-seq-identified ORFs) (Tables 1 and 2).

**TABLE 1:** List of sORFs

Ribo-seq-identified sORF	Coordinates	Upstream flanking gene	Downstream flanking gene	Strand	Expression (highest peak)	Start codon	Nucleotide sequence	Peptide sequence	Small protein length (amino acids)	Theoretic mol wt (kDa)^*a*^	Presence of secretion signal^*b*^	Found within/overlapping sRNA^*c*^
*rio1*	24644–24742	*spv_0025*	*spv_0027*	+	48	CTG	CTGCGTGAAGCGGGTCAGGGGAGGAATCCAGCAGCCCTAAGCGATTTGAATTGTGTGCTCTTTTTTTCGTGCTTTTTCCGAATAAATAAGATAGAATAA	MREAGQGRNPAALSDLNCVLFFSCFFRINKIE	32	3.6	No	scRNA
*rio2*	29719–29778	*spv_0033*	*spv_0034*	+	6.25	ATG	ATGACTGTACGTCATCAGAAGTTTCAGCGACCATCATTTTTGAACAGTGATAGCACTTGA	MTVRHQKFQRPSFLNSDST	19	2.2	No	*srf-01*
*rio3*	39961–39996	*spv_0047*	*spv_2085*	+	53	ATG	ATGAATCGTAATTTAGAACGGTGTTATCTATTCTGA	MNRNLERCYLF	11	1.4	No	*srf-02*
*rio4*	39885–39971	*spv_0047*	*spv_2084*	+	24.4	ATG	ATGATGAATCTGAATCAAAATTATTTTGCGCATGTAAAGAGGAGTCTTATAGTAACGAGTCAAAAAAGGAGTAACTATGAATCGTAA	MMNLNQNYFAHVKRSLIVTSQKRSNYES	28	3.3	Yes	
*rio5*	78879–78992	*spv_0077*	*spv_2092*	+	2.7	ATG	ATGAAAATCAAAGATCAAACTAGGAAACTAGCTACGGGCTGCTCAAAACACTGTTTTGAGGTTGCAGATAGAACTGACGAAGTCAGTAACATCTATACGGCAAGGCGACGTTGA	MKIKDQTRKLATGCSKHCFEVADRTDEVSNIYTARRR	37	4.3	No	spd sr8
*rio6*	86107–86193	*spv_0082*	*spv_0083*	+	10	ATG	ATGAAGCTCGTCAACAGGTGTCTTATGACAAGTAACCTTGGCTGTTTAGGCGAAGGGCATCTGCACGAATCAGGGCTTTCTAAGTGA	MKLVNRCLMTSNLGCLGEGHLHESGLSK	28	3	No	
*rio7*	113831–113908	*spv_0112*	*spv_0113*	+	13	TTG	TTGTTAAAGGAGGCTCAGTCCTTGGGCAAAAATCTGCTGTACTACAATGCTTGGAAAGATATCAAGAACAAAGAATGA	MLKEAQSLGKNLLYYNAWKDIKNKE	25	2.9	No	
*rio8*	113958–113987	*spv_0112*	*spv_0113*	+	5.4	TTG	TTGGGTGACATCAAAGAAAAGTTAGATTAA	MGDIKEKLD	9	1	No	
*rio9*	113770–113814	*spv_0112*	*spv_0113*	+	4.2	TTG	TTGATTAGTCTGGCTATAATCTTTCCATGGGGCTGGCCGATATAA	MISLAIIFPWGWPI	14	1.6	No	
*rio10*	118625–118672	*spv_0116*	*spv_2099*	+	3.8	ATG	ATGTGTAGGAATAGCCCCTTTTTTTCACGTATATATAATAGGTTCTGA	MCRNSPFFSRIYNRF	15	1.9	No	
*rio11*	118721–118750	*spv_0116*	*spv_2099*	+	2.5	TTG	TTGTATTTTAACTATTGCTCGAATTTATAG	MYFNYCSNL	9	1.1	No	
*rio12*	119733–119777	*spv_0116*	*spv_2099*	+	40	TTG	TTGATTAGTCTAGCTATAATCTTTCCATGGGGCTGGTCGATATAA	MISLAIIFPWGWSI	14	1.6	No	
*rio13*	122716–122766	*spv_0119*	*spv_0120*	+	15	ATG	ATGGCTGATGAATCTTATTTATACTGTTGCAGTAGGCATTGTCTAGGATAA	MADESYLYCCSRHCLG	16	1.8	No	
*rio14*	122780–122845	*spv_0119*	*spv_0120*	+	8	ATG	ATGGGGGAAATTGGAATAATTTTAGTAGAAACATTAAAGGATTATGGACAAGTAGCGCAGGATTAG	MGEIGIILVETLKDYGQVAQD	21	2.2	No	
*rio15*	127035–127055	*spv_0124*	*spv_2103*	+	57.5	ATG	ATGGGGATTGTATTACTCTGA	MGIVLL	7	0.64	No	
*rio16*	132259–132294	*spv_0127*	*spv_0128*	+	25	ATG	ATGTTGCAGGCTTTTTTGTCCTGCACTTCTTTGTAG	MLQAFLSCTSL	11	1.2	No	*srf-04*
*rio17*	149686–149748	*spv_0143*	*spv_0144*	−	2.1	ATG	ATGAGGTGTGAGCTCAAAATATCCTCCAGTTATGTTTTTCCTAATAGTATACCGGAAGAGTGA	MRCELKISSSYVFPNSIPEE	20	2.3	No	*srf-05*
*rio18*	165545–165586	*spv_0160*	*spv_0161*	+	12.5	TTG	TTGAAGTTGTTCAACTGTTTTTTGAGTATAAACAGTCTTTAA	MKLFNCFLSINSL	13	1.5	No	
*rio19*	174277–174363	*spv_0169*	*spv_0170*	−	2.2	ATG	ATGAAAGAAGATAAAAGTATATTTGTGCTTTTTGCGTGCTCTGAAATGATTACTTGTCATTTCAGAGCATTTTTGTTAATCGCATAA	MKEDKSIFVLFACSEMITCHFRAFLLIA	28	3.2	No	
*rio20*	186024–186104	*spv_0182*	*spv_0183*	+	105	CTG	CTGGGAGACTGTATCAGCCCAACTCCCAGAAATGGGGAAGGGTTGGAACGTCCAGTGGATGTTTTTAGCCTAGCTCTTTGA	MGDCISPTPRNGEGLERPVDVFSLAL	26	2.7	No	
*rio21*	186133–186174	*spv_0182*	*spv_0183*	+	33	ATG	ATGTTTTACTCCACGTTAATATTCATAGTTGCTAGTGATTAG	MFYSTLIFIVASD	13	1.5	No	
*rio22*	185918–185992	*spv_0182*	*spv_0183*	+	12.5	CTG	CTGGTAAGTCCAGTGGACGTTTTTAGCCTGACGCTAAAAATAAAAACCGTCAGTAAGTTCGGGGGACGTTTTTAG	MVSPVDVFSLTLKIKTVSKFGGRF	24	2.6	No	
*rio23*	188045–188092	*spv_0184*	*spv_0185*	+	5.75	ATG	ATGCAAGTAGAAAGGGCTGGATTTTTCAGCCTTTTTACTTTTACCTAG	MQVERAGFFSLFTFT	15	1.7	No	
*rio24*	195914–195985	*spv_0191*	*spv_0192*	+	5.5	TTG	TTGCGACACGCTCGGTTGCATTGCCACGCAACACCGCGTCGGTTTTCTTGTGGAGCTAGCCTATTATCTTAA	MRHARLHCHATPRRFSCGASLLS	23	2.6	Yes	
*rio25*	195901–195972	*spv_0191*	*spv_0192*	+	20	TTG	TTGATGCAAGAGGTTGCGACACGCTCGGTTGCATTGCCACGCAACACCGCGTCGGTTTTCTTGTGGAGCTAG	MMQEVATRSVALPRNTASVFLWS	23	2.5	No	
*rio26*	202954–203037	*spv_0206*	*spv_0207*	+	40	TTG	TTGACGAAGAAACTAAAGTTTCTAGGAAAGTTTATCTTTTTCACACAGAGTTTAGCCCGGGTTCAATTGGGCTTGCCAATTTGA	MTKKLKFLGKFIFFTQSLARVQLGLPI	27	3.1	Yes	
*rio27*	203084–203146	*spv_0206*	*spv_0207*	+	8.8	TTG	TTGCTTCTGCATTCAATTGTCTATTTTTGCTCGTGCTGTTACGCTCTTTGTATCATGTATTAA	MLLHSIVYFCSCCYALCIMY	20	2.3	No	
*rio28*	249098–249157	*spv_0252*	*spv_0253*	+	1.68	ATG	ATGAATACAAGGAGTTTTATCTTTTTCGCGCAGCATCCCGTTCCAGCTCATATCGGCTAA	MNTRSFIFFAQHPVPAHIG	19	2.1	No	
*rio29*	272815–272874	*spv_0273*	*spv_0274*	+	11.3	TTG	TTGAAAGGCCCCGGAACCTTCCAAATACTTTTCGATGGGAAGGAACACCCATCACCGTAA	MKGPGTFQILFDGKEHPSP	19	2	No	
*rio30*	306391–306480	*spv_0307*	*spv_0308*	+	18	ATG	ATGTTTCATCTAGAAATCTTCAGAAGTAAAGATAGTCTACTCCTGCTTGAAAAAGAAAAACCGGAAATAGTACATAGAGTAGCGATTTAG	MFHLEIFRSKDSLLLLEKEKPEIVHRVAI	29	3.4	No	
*rio31*	400274–400312	*spv_0398*	*spv_0399*	+	28	ATG	ATGGTAAAATCCAATGTAAAAATCATTCTCAGCTATTGA	MVKSNVKIILSY	13	1.3	No	
*rio32*	402069–402098	*spv_0399*	*spv_0400*	+	11.9	ATG	ATGGTTTTACGAACGAATAGGCGAAAATAA	MVLRTNRRK	9	1.1	No	
*rio33*	401926–401958	*spv_0399*	*spv_0400*	+	11	TTG	TTGTTGGACGCTAGTCGCTATTTGGCGAACTAG	MLDASRYLAN	10	1.1	No	
*rio34*	408631–408684	*spv_0406*	*spv_2159*	+	18	ATG	ATGAAAAAGATTATGAGAAAAATTGCATCGTTATTATTGGTTCTAGTTGTATAA	MKKIMRKIASLLLVLVV	17	1.9	Yes	
*rio35*	411179–411250	*spv_0409*	*spv_0410*	+	6.1	ATG	ATGAATTTGAAGGACATAAGGAATACCTATCTCTCAGATGATTTATTGAGGAAGAAAGATAGGAGTTTTTGA	MNLKDIRNTYLSDDLLRKKDRSF	23	2.8	No	
*rio36*	444912–444977	*spv_0441*	*spv_0442*	+	12.5	CTG	CTGGTGCAGTCGTCCCAGATTATTCTTATTAGTAGGGTCTTGTTTTCTATATCCCCTCGTAGTTAA	MVQSSQIILISRVLFSISPRS	21	2.3	No	
*rio37*	485231–485263	*spv_0474*	*spv_0475*	+	3.5	TTG	TTGGAAAGGCTAGAACTAAAGAATGACGTGTAG	MERLELKNDV	10	1.2	No	
*rio38*	497485–497523	*spv_0490*	*spv_0491*	+	10	ATG	ATGCGGGCTTGGCCCGAAATTGGGTGGTACCGCGGATAA	MRAWPEIGWYRG	12	1.5	No	
*rio39*	508756–508830	*spv_0500*	*spv_0501*	+	30	ATG	ATGTTATCACTAATACAAGTGAGCAGGAACCTATTTAATCACATCAGAAGAAGTTTCTTGATGTTTTTTAAGTAG	MLSLIQVSRNLFNHIRRSFLMFFK	24	2.9	No	*srf-10*
*rio40*	565754–565780	*spv_0553*	*spv_2193*	+	17	ATG	ATGTATAGTAGACTGAATCTAAAATAG	MYSRLNLK	8	1	No	
*rio41*	569211–569276	*spv_0556*	*spv_0557*	+	24	ATG	ATGATGTTTGTAATTGAAGAAGTCAAGGATGAAAATCAAAAAAGGCAGTTGTCGCTGAGGTTTTGA	MMFVIEEVKDENQKRQLSLRF	21	2.6	No	
*rio42*	569735–569764	*spv_0557*	*spv_2196*	+	9.4	TTG	TTGGTAGAGCAAGGCACTCGTAAAGCCTAG	MVEQGTRKA	9	1	No	
*rio43*	616529–616573	*spv_0590*	*spv_0510*	+	4.4	ATG	ATGAAGCTATTGTTTTATAGTATAATTAATTTGTATAAAATTTAA	MKLLFYSIINLYKI	14	1.7	No	
*rio44*	640777–640818	*spv_0619*	*spv_0620*	+	2.3	ATG	ATGGGAGTATCGCAAAAAATGACTCATCGTATTCAATTTTGA	MGVSQKMTHRIQF	13	1.5	No	
*rio45*	653885–653935	*spv_0632*	*spv_0633*	−	11.8	ATG	ATGGAAGTGGAAATGATAATGGGGACTAGCAGTCTTCTATTGCCTTTCTAA	MEVEMIMGTSSLLLPF	16	1.7	No	
*rio46*	657363–657389	*spv_0635*	*spv_0636*	+	275	TTG	TTGGAGAACGTTTCCAATTCTATGTAA	MENVSNSM	8	0.9	No	
*rio47*	669265–669354	*spv_0649*	*spv_0650*	+	1.3	ATG	ATGACTTGGAAAAGTATTTCCAGTCACGAAAGGAGGTTGGGTTTTTGTTTCTGTCTAATGAAAGCAGAGCAAAAATTTGACCTTTTTTGA	MTWKSISSHERRLGFCFCLMKAEQKFDLF	29	3.5	No	
*rio48*	676352–676426	*spv_0657*	*spv_0658*	+	182	ATG	ATGGACATTTCAGTAATTCGTCAGAAAATTGACGCAAATCGTGAAAAATTAGCTTATTTCAGGGGGTCTCTTTGA	MDISVIRQKIDANREKLAYFRGSL	24	2.8	No	
*rio49*	684026–684079	*spv_0663*	*spv_0664*	+	750	ATG	ATGGTAGGTATTTATTACGAAGAGTTTTCCTATCAGTACTTTGTAACTCTATAA	MVGIYYEEFSYQYFVTL	17	2.1	No	
*rio50*	738388–738480	*spv_0722*	*spv_0723*	+	1.4	CTG	CTGGAAAGAGATGAACAAATCAAATACATAAAAGATAAACTCATTTTTATTCGTTTGGTAAAAATAAAATGCATATTTTTAAAGAAAGATTGA	MERDEQIKYIKDKLIFIRLVKIKCIFLKKD	30	3.7	No	
*rio51*	764625–764684	*spv_0750*	*spv_0751*	+	2	ATG	ATGGTATTGATCTTGATAAAATTTTTAAAATACTGTCATTTTGAATATAAAGGAGTTTGA	MVLILIKFLKYCHFEYKGV	19	2.3	No	
*rio52*	767622–767705	*spv_0756*	*spv_0757*	+	20	TTG	TTGAAAGACGTGAATGATATGAACATGTCCTTGCTGGTGCTTAGGAAAAAAATTATAAGTATGTCAAGTTTAAGAAAAACTTGA	MKDVNDMNMSLLVLRKKIISMSSLRKT	27	3.1	Yes	
*rio53*	773033–773068	*spv_0761*	*spv_0762*	+	57.5	TTG	TTGCTCTATTTCTGGGGAAATCAGACGTTTTTCTAG	MLYFWGNQTFF	11	1.4	No	
*rio54*	863206–863247	*spv_0846*	*spv_0847*	+	9.6	CTG	CTGGCCCTACGGATGAAAAGTTTCGAAGAAACGCTATCATAA	MALRMKSFEETLS	13	1.5	No	
*rio55*	865083–865118	*spv_0850*	*spv_0851*	+	14	TTG	TTGTGTTGTACGATTTTAACTGAGGCCTTGCACTAG	MCCTILTEALH	11	1.2	No	
*rio56*	1051996–1052016	*spv_0898*	*spv_0900*	−	200	TTG	TTGAGCATAAGGAGGTCATAA	LSIRRS	6	0.7	No	*srf-17*
*rio57*	1036860–1036916	*spv_0915*	*spv_2289*	−	9.9	CTG	CTGTATCTATTGACAATGATAATTATTATCGATACAATAGACTTGAAATATGTTTAA	MYLLTMIIIIDTIDLKYV	18	2.1	No	
*rio58*	962247–962309	*spv_0992*	*spv_0991*	−	14.35	TTG	TTGAACCTTTTATCCCGAACCTTGAAATGTAAAGGTGAGGAAGCTAGAAACAGCTTAAAATAA	MNLLSRTLKCKGEEARNSLK	20	2.2	No	
*rio59*	958487–958513	*spv_0997*	*spv_0996*	−	6.7	TTG	TTGGAAGAAATAGCGTTTTTAACTTGA	MEEIAFLT	8	0.95	No	
*rio60*	921151–921201	*spv_1017*	*spv_1018*	−	13.8	ATG	ATGAAGAACAAACCAAGATTCAAGCAGGAATTCCTACTGATAATGAAGTAA	MKNKPRFKQEFLLIMK	16	2	No	
*rio61*	917345–917374	*spv_1021*	*spv_1023*	−	25.5	ATG	ATGAAAAAGAAAACAATAGCAATTATATAG	MKKKTIAII	9	1	No	
*rio62*	1085315–1085347	*spv_1059*	*spv_1060*	−	1.6	ATG	ATGCCTTTCCTTGTTCACAGGAAATTTATATAA	MPFLVHRKFI	10	1.2	No	
*rio63*	1153159–1153251	*spv_1121*	*spv_2314*	−	5.5	ATG	ATGATGATACTTTTCGAAAATCTCTTCAAACTACGTCAGCTCAGCTTTGCCTTGCTGTGTTTTGAGCAAGCTACGGTTAGCTTCCGAGTTTGA	MMILFENLFKLRQLSFALLCFEQATVSFRV	30	3.5	No	
*rio64*	1189201–1189269	*spv_1159*	*spv_1158*	−	20	ATG	ATGGCTATAGTTGAAATTATAAATCTAACAAAAAGCTTTAAAGATATTGAAGTTATTCATAACACTTAA	MAIVEIINLTKSFKDIEVIHNT	22	2.5	No	
*rio65*	1190190–1190255	*spv_1160*	*spv_1161*	−	1.83	ATG	ATGAAAGATAGTTATTGGTTAAATTATTTTCCGGAGTATAGTTTAGAAACGTTTGAAGTAGAATAG	MKDSYWLNYFPEYSLETFEVE	21	2.6	No	
*rio66*	1242216–1242290	*spv_1212*	*spv_1213*	+	2	ATG	ATGTGTAAGAATCACAATAAAAAATGCTCTTCCGTCTTGGAGGAGCATTTCTTTTTATCAACGAAAATCAAATAG	MCKNHNKKCSSVLEEHFFLSTKIK	26	2.8	No	
*rio67*	1242341–1242406	*spv_1212*	*spv_1213*	+	0.9	TTG	TTGTATCAGCAATATGTGTCTGTCAAATTTAGTGACAAAGGTAGTAGAAGAAAGATGAAGAAATAA	MYQQYVSVKFSDKGSRRKMKK	21	2.5	No	
*rio68*	1250407–1250430	*spv_1217*	*spv_1218*	−	1.75	ATG	ATGGCTTGGATCGACAATACCTAA	MAWIDNT	7	0.8	No	
*rio69*	1250035–1250073	*spv_1217*	*spv_1218*	−	2	TTG	TTGAATGAAGGTGGTACCGCGGTTTTTCGCCCTTCGTGA	MNEGGTAVFRPS	12	1.2	No	
*rio70*	1264626–1264679	*spv_1232*	*spv_2332*	−	18	ATG	ATGGGGGAAGGGATAGACAAGAGATTTTATCCACATATGAAAAAAGGAGGTTAG	MGEGIDKRFYPHMKKGG	17	1.9	No	
*rio71*	1274211–1274282	*spv_1245*	*spv_1246*	−	5.4	ATG	ATGGAAGAAAAAATCAAAATTAAACCGCATTTTTGCTTGACAATTATTCCTTTTACGTGTAGAATGAAATAG	MEEKIKIKPHFCLTIIPFTCRMK	23	2.8	No	
*rio72*	1277771–1277803	*spv_1248*	*spv_2334*	−	1.4	ATG	ATGATGACGCTGACAATGGGAGTATTATTGTAA	MMTLTMGVLL	10	1.1	No	
*rio73*	1287975–1288013	*spv_1258*	*spv_1259*	+	32	ATG	ATGACCGAATTAGAAAGAAAAAATCGAAAAATTAGCTAA	MTELERKNRKIS	12	1.5	No	
*rio74*	1297923–1297994	*spv_1267*	*spv_2337*	+	9	ATG	ATGATATGGGATTTTCATATAATAAATTGTAACCGCCCAATAACGAAGTCTATTGAAAAATCTCCAGATTAG	MIWDFHIINCNRPITKSIEKSPD	23	2.7	No	
*rio75*	1357604–1357621	*spv_1342*	*spv_1343*	−	5	ATG	ATGTTGTTGATATTTTAA	MLLIF	5	0.6	No	
*rio76*	1357729–1357752	*spv_1342*	*spv_1343*	−	30	ATG	ATGTATATGAGTAACTATCGATAA	MYMSNYR	7	0.9	No	
*rio77*	1404509–1404604	*spv_1383*	*spv_1384*	−	27	CTG	CTGGCAGAAACCTGTGATAGTGTCGTCATTCCGAATTTTATGCTGAAAAGTATGCTTTCCGGCCCTATCTTAAACAGCGAGACTTGTTATGATTAA	MAETCDSVVIPNFMLKSMLSGPILNSETCYD	31	3.4	No	
*rio78*	1433395–1433523	*spv_1413*	*spv_1414*	−	5.4	TTG	TTGCTCTGGTCTTGTGTTATACTAGATAGGTTGCAAAGAAAACAGTACTTTTCTTTTGTGGAAAAGAAGCAACACGATTTTATATCTAGTTATATGAAAATACGTCATAAAAAGAAAAGTATAAACTAA	MLWSCVILDRLQRKQYFSFVEKKQHDFISSYMKIRHKKKSIN	42	5.2	No	
*rio79*	1456875–1456949	*spv_1438*	*spv_1439*	−	117	ATG	ATGCTTTCCTACGTTCGACATTACCCACTAGCGATAGCTAAATTAATGTGTCTGTGCTCTCCTAAAATCTGCTGA	MLSYVRHYPLAIAKLMCLCSPKIC	24	2.7	Yes	
*rio80*	1457861–1457905	*spv_1439*	*spv_1440*	−	14.9	TTG	TTGGTTACAGGCATGCCAACCTGTCACTCGGATGAAGCCAAATAA	MVTGMPTCHSDEAK	14	1.5	No	
*rio81*	1480252–1480278	*spv_1464*	*spv_1465*	+	1.6	TTG	TTGAAACGGAGGATTTTTGAATATTAG	MKRRIFEY	8	1.1	No	
*rio82*	1528556–1528618	*spv_1506*	*spv_1507*	−	120	ATG	ATGACAGTAACGATTAAAGTAAATTACCAAACCACTTTCCAAAAGAAGGAAGCAAAAAACTAG	MTVTIKVNYQTTFQKKEAKN	20	2.3	No	*srf-21*
*rio83*	1539897–1539920	*spv_1517*	*spv_1518*	−	775	ATG	ATGATATACCATCGTTTAGAATAA	MIYHRLE	7	0.9	No	
*rio84*	1539963–1540064	*spv_1517*	*spv_1518*	−	358	ATG	ATGGGCTTTAAAAAATATTTGAAGAATTTACCGAAAAACTCTGGATTTTTGATTTGGAGTTGGATTCAACTTATCTGGTTTGAAACATGGTTTTGGGGATAA	MGFKKYLKNLPKNSGFLIWSWIQLIWFETWFWG	33	4.1	Yes	
*rio85*	1579688–1579789	*spv_1558*	*spv_1559*	−	140	CTG	CTGAATTTGGGCGAGCAAGGCGAGCCCCATAGAGAATACTTTTCGCTGTGGTGTAAGTTGGTACAAGTGATTGTACCAACTGCGGAAAATTTGAGACCTTAG	MNLGEQGEPHREYFSLWCKLVQVIVPTAENLRP	33	3.8	No	
*rio86*	1673721–1673771	*spv_1661*	*spv_1662*	−	16	TTG	TTGACATTCTATCAAGCTGTCGGTCAGTTCGTTCAGTACAAGGAATCATAA	MTFYQAVGQFVQYKES	16	1.9	No	
*rio87*	1721860–1721904	*spv_1725*	*spv_1726*	−	5.6	ATG	ATGCTTGCGACAAAAAGAGGCGATGATCTCTCTGCGGATATCTGA	MLATKRGDDLSADI	14	1.5	No	
*rio88*	1731142–1731255	*spv_1737*	*spv_1738*	−	46	ATG	ATGAAAAGGACATATAGAGACTGTAAAAATATACTTTTGAAAAGCTTTTTAGTCTGGGGTGTTATTGTAGATAGAATGCAGACCTTGTCAGTCCTATTTACAGTGTCAAAATAG	MKRTYRDCKNILLKSFLVWGVIVDRMQTLSVLFTVSK	37	4.3	Yes	
*rio89*	1786300–1786332	*spv_1790*	*spv_1791*	−	5	TTG	TTGCATTCGAAAAAGCTGGAAACATTTGCCTAG	MHSKKLETFA	10	1.1	No	
*rio90*	1796868–1796975	*spv_1803*	*spv_1804*	−	9.3	TTG	TTGGTTCTCTCTTTTTTGATTTTCAGTAATTCATTTTGGCAGCGTATACTTTTTGTCTCCAGTCTTTTCAATAAATACCGTAGTTTAGGTATACACATTGAAATTTAA	MVLSFLIFSNSFWQRILFVSSLFNKYRSLGIHIEI	35	4.2	No	
*rio91*	1814148–1814210	*spv_1828*	*spv_1829*	−	2.8	ATG	ATGAATTTCAATCTCCCAATTTATTTGTTCAAATATTGTTTTAATATATTGAATAAATTCTGA	MNFNLPIYLFKYCFNILNKF	21	2.5	No	
*rio92*	1814385–1814408	*spv_1828*	*spv_1829*	−	1.6	ATG	ATGTTGAAAAATATCTTCGTATAG	MLKNIFV	7	0.8	No	
*rio93*	1858646–1858711	*spv_1878*	*spv_1879*	−	41.08	TTG	TTGTTATTCTTCGTTCCTTTTTTATATTATTTTTGGTATAATTATAGTTATTCAAATTTTATTTAG	MLFFVPFLYYFWYNYSYSNFI	21	2.8	No	
*rio94*	1907589–1907618	*spv_1933*	*spv_1934*	−	29.8	ATG	ATGCTTGAAAAGGAGTATACTTATAAGTAA	MLEKEYTYK	9	1.2	No	
*rio95*	2006759–2006857	*spv_2027*	*spv_2028*	−	37	CTG	CTGAGAGGAAGTGTTAAACTTCGACCGCACCTGATCTGGGTAATGCCAGCGGAGGGAACGATACTTAGTCTAATTTTGCACCTTTTCCATGTATGGTAA	MRGSVKLRPHLIWVMPAEGTILSLILHLFHVW	32	3.7	No	
*rio96*	2012589–2012618	*spv_2032*	*spv_2033*	−	5	ATG	ATGTGTAAAGGTAGGTTTACTGAATTGTAA	MCKGRFTEL	9	1	No	
*rio97*	105596–105616	*spv_2096*	*spv_0106*	+	425	ATG	ATGGACTACGATACTTGTTGA	MDYDTC	6	0.7	No	
*rio98*	120796–120822	*spv_2100*	*spv_0118*	+	15	ATG	ATGAGGTTGGGAAAAAACTCAATTTGA	MRLGKNSI	8	0.9	No	
*rio99*	120952–120984	*spv_2100*	*spv_0118*	+	5.5	ATG	ATGACTTTATTTAGGTGTTTGACATTACTATAG	MTLFRCLTLL	10	1.2	No	
*rio100*	121373–121429	*spv_2101*	*spv_0119*	+	8.5	ATG	ATGATGACTAAAGTTTTTATCAATAATTTGGGCTCCTTGTCAACTGTAGTGGGTTGA	MMTKVFINNLGSLSTVVG	18	1.9	Yes	
*rio101*	141806–141937	*spv_2113*	*spv_2115*	+	38	ATG	ATGAGTTGGTTAGACGCTTTTCATTATAGGTCATATGGGGCTTTTTTCTACAAGAAACGACCCTATAATTCCTGGGGTGGGATTACCCACTACAGAAATTATAGAGCCAAAGCATTCCAAAGTCTTGTCTGA	MSWLDAFHYRSYGAFFYKKRPYNSWGGITHYRNYRAKAFQSLV	43	5.2	No	
*rio102*	420072–420098	*spv_2164*	*spv_0420*	+	2	CTG	CTGTTACTAGAAAAAAGAGGACATTAA	MLLEKRGH	8	0.9	No	
*rio103*	742170–742214	*spv_2226*	*spv_2227*	+	55	ATG	ATGAAAATTGGTCAACGAATTATGCGCTTTGGCATAAAAAATTAA	MKIGQRIMRFGIKN	14	1.6	No	
*rio104*	811459–811533	*spv_2240*	*spv_0792*	+	27	ATG	ATGAACACATTAAATGAGAAAGTAATCAATATCTGTAAAGCAGTAGTTAAAGAAACTTTAATCCAAGACATTTAG	MNTLNEKVINICKAVVKETLIQDI	24	2.7	No	
*rio105*	849735–849788	*spv_2251*	*spv_0833*	+	35	CTG	CTGATTGGCTTTTTCAATGTGAATCTTAACTTCATACTCCCAAAGAGGTATTAG	MIGFFNVNLNFILPKRY	17	2	No	
*rio106*	1032903–1032932	*spv_2288*	*spv_0918*	−	12	ATG	ATGGCAAATACAGCACAGAATTTAAGATAA	MANTAQNLR	9	1	No	
*rio107*	1037861–1037899	*spv_2291*	*spv_0914*	−	9.2	TTG	TTGTTGGTTTCGTGTCATAACAGTTATAGAGGCAAATAG	MLVSCHNSYRGK	12	1.3	No	
*rio108*	1037928–1037963	*spv_2291*	*spv_0914*	−	7.9	ATG	ATGATAAAGTTTGTGAATATCTTAGTCCTCATTTGA	MIKFVNILVLI	11	1.3	No	
*rio109*	1038016–1038045	*spv_2291*	*spv_0914*	−	18	ATG	ATGATTGATAAAGGCAACAAAAAATTTTAG	MIDKGNKKF	9	1	No	
*rio110*	1278108–1278194	*spv_2334*	*spv_1249*	−	9.1	ATG	ATGAGTGAAAATTATCAAGTTGGAATGTTTGTATCTAAATATATTAGCATGTATTTAGATAAGATGTCTGCAATCCTTTATATATGA	MSENYQVGMFVSKYISMYLDKMSAILYI	28	3.3	No	
*rio111*	1469164–1469196	*spv_2369*	*spv_2370*	−	6.8	ATG	ATGGAAAGAGGAACGAATGAAGATGAAAGCTAG	MERGTNEDES	10	1.1	No	
*rio112*	1619475–1619567	*spv_2394*	*spv_1605*	−	4.9	TTG	TTGAGGTGGCACCGCGTTACCAACGCCCTCACACGGAAGTATATTCTGTGTGTGGGCTTTTTTCTATCCGTCGTTTGGTTTATCTTTTATTAG	MRWHRVTNALTRKYILCVGFFLSVVWFIFY	30	3.7	Yes	
*rio113*	1619912–1619941	*spv_2394*	*spv_1605*	−	12.5	ATG	ATGGAGTGTTCTAAAATAAGTTCTGTTTAG	MECSKISSV	9	0.9	No	
*rio114*	1751386–1751403	*spv_2420*	*spv_1757*	+	1.2	ATG	ATGTTGTTTCAGTATTGA	MLFQY	5	0.7	No	

a See reference 65.

b See reference 66.

c See references 2 and 3. scRNA, small cytoplasmic RNA.

**TABLE 2:** sRNAs involved in virulence

sRNA^*a*^ in Tigr4	Tigr4 flanking gene	Tigr4 flanking gene	sRNA D39 homolog	Host	Fitness (<1, fitness defect)	sORF	Expression (rpm)
F38	*sp_1012*	*sp_1013*	*srf-17*	Nasopharynx	0	*rio56*	115
SN39	*sp_0761*	*sp_0762*		Nasopharynx	0	*rio49*	550
F52	*sp_0041*	*sp_0042*	*srf-02*	Nasopharynx	0	*rio3*	208
trn0760	*sp_1625*	*sp_1626*		Nasopharynx	0	*rio79*	73

a See references 1 and 67.

**FIG 2:**
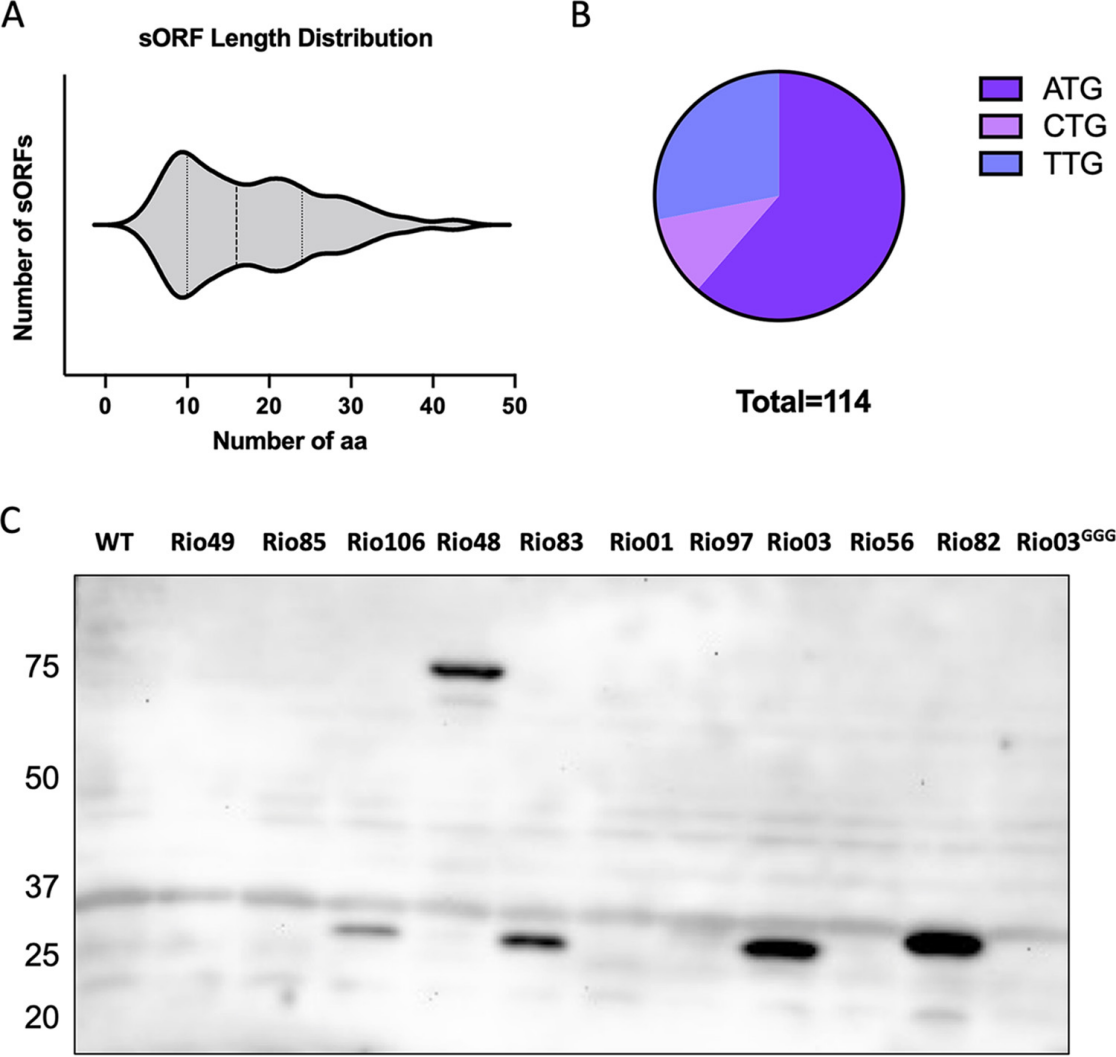
Identification and validation of unannotated sORFs. (A) Violin plot showing the distribution of protein lengths (amino acids [aa]) encoded by the 114 sORFs identified. (B) Start codon identity distribution of the sORFs. (C) Western blotting of C-terminally sfGFP-tagged sORFs expressed from their native locus.

The identified sORFs range in length from 5 to 43 amino acids, with the majority having an AUG start codon (Fig. 2A and B). Using tBLASTn analysis (33), we investigated the conservation of the sORFs among six clinically relevant S. pneumoniae serotypes (1, 4, 14, 19A, 23F, and 19F). We found that 49 sORFs were conserved in all six serotypes, with the remaining sORFs being conserved in at least one of the serotypes except for *rio34*, *rio72*, and *rio89* (see Table 3). We also identified 10 sORFs encoding proteins containing putative signal peptides as determined by SignalP analysis (34) suggested their insertion into or translocation across the cytoplasmic membrane (Table 1). One of these, *rio84*, was found adjacent to an Rgg family member gene, and we hypothesized that it encodes the signaling peptide for a pheromone receptor QS system (see below). Nine sORFs are located within or overlap the previously annotated noncoding RNAs (ncRNAs), two of which were previously associated with fitness defects determined by transposon-insertion sequencing (TIS) (Tables 1 and 2) (1, 3).

To validate the Ribo-RET results and demonstrate sORF translation, 6 sORFs displaying the highest number of read counts at start codons (*rio48*, *rio49*, *rio83*, *rio85*, *rio97*, and *rio106*) and 4 sORFs located within documented ncRNAs (*rio01*, *rio3*, *rio82*, and *rio56*) were selected to be tagged with translation reporters (Table 1). A sequence encoding superfolder green fluorescent protein (*sfGFP*) (lacking its own start codon) was placed at the 3′ end of each selected sORF at its native chromosomal locus to generate in-frame translational fusions. If translated, the addition of *sfGFP* should increase the molecular weight of each sORF peptide by ~27 kDa. Cells containing the tagged constructs were cultured to mid-log phase in chemically defined medium (CDM) to mimic the conditions used in ribosome profiling experiments, and the expressed proteins were evaluated by Western blotting using an anti-GFP antibody. Of the 10 sfGFP-tagged constructs, 5 produced a strong band with the expected mobility on an SDS gel, verifying their translation (Fig. 2C). To demonstrate that sfGFP was not independently translated when placed in frame with sORFs, the start codon of the sORF *rio3* fused to *sfGFP* was mutated from ATG to GGG. The production of the fusion protein was completely abolished, demonstrating not only the translation of the identified sORFs but also the accuracy of mapping its start codon by Ribo-RET/Ribo-LEF.

Unexpectedly, *rio48*::*sfGFP*, located immediately upstream of the gene encoding peptide release factor 2 (RF2), *prfB*, produced a strong band of ~70 kDa. In E. coli, the expression of RF2 is autoregulated by programmed frameshifting; RF2 deficiency stimulates a +1 frameshift resulting in the readthrough of the in-frame UGA stop codon and the translation of the full-size functional RF2 protein (35). In E. coli, previous studies have demonstrated that the frameshift mechanism exploits several key features of the *prfB* mRNA: a Shine-Dalgarno (SD) sequence 3 nucleotides upstream of the frameshift site (AGG GGG), the frameshift site (CUU UGA), and the context of the UGA stop codon flanked with a 3′ C (Fig. S4) (36–38). The short distance between the SD sequence and the frameshifting site creates tension destabilizing the interactions between the P-site and the anticodon of the ribosome, resulting in a +1 frameshift. Furthermore, the genetic context of the UGA stop codon in proximity to a C nucleotide has been demonstrated to be the least efficient termination signal (37–39). These key mRNA features are also conserved in *rio48*, suggesting that *prfB* in S. pneumoniae is regulated in a similar manner. Likewise, a +1 frameshift at the UGA stop codon of *rio48* is in frame with downstream *prfB*, and therefore, programmed frameshifting during Rio48 translation could stimulate the expression of RF2. The *rio48*::*sfGFP* construct retains the UGA stop codon of *rio48* after sfGFP and likely results in readthrough and the generation of the larger gene product corresponding to ~70 kDa seen on the immunoblot. Thus, in this instance, Ribo-RET likely identified the correct translation start site for *prfB*.

10.1128/mbio.01247-22.4 FIG S4 Sequence alignment of *prfB* in E. coli and S. pneumoniae D39. Shown is a schematic representation of *prfB* in E. coli and S. pneumoniae. The stop sign represents the internal UGA stop codon where a +1 frameshift occurs. The mRNA sequence is of the 5′ end of *prfB*, and the underlined sequence depicts the conserved sequence in both bacteria. SD highlights the Shine-Dalgarno sequence, FS highlights the frameshift site, and the asterisk identifies the UGA stop codon. Download FIG S4, TIF file, 1.8 MB. Copyright © 2022 Laczkovich et al. 2022 Laczkovich et al. https://creativecommons.org/licenses/by/4.0/ This content is distributed under the terms of the Creative Commons Attribution 4.0 International license.

### Peptides associated with an Rgg-type quorum sensing system.

**FIG 3:**
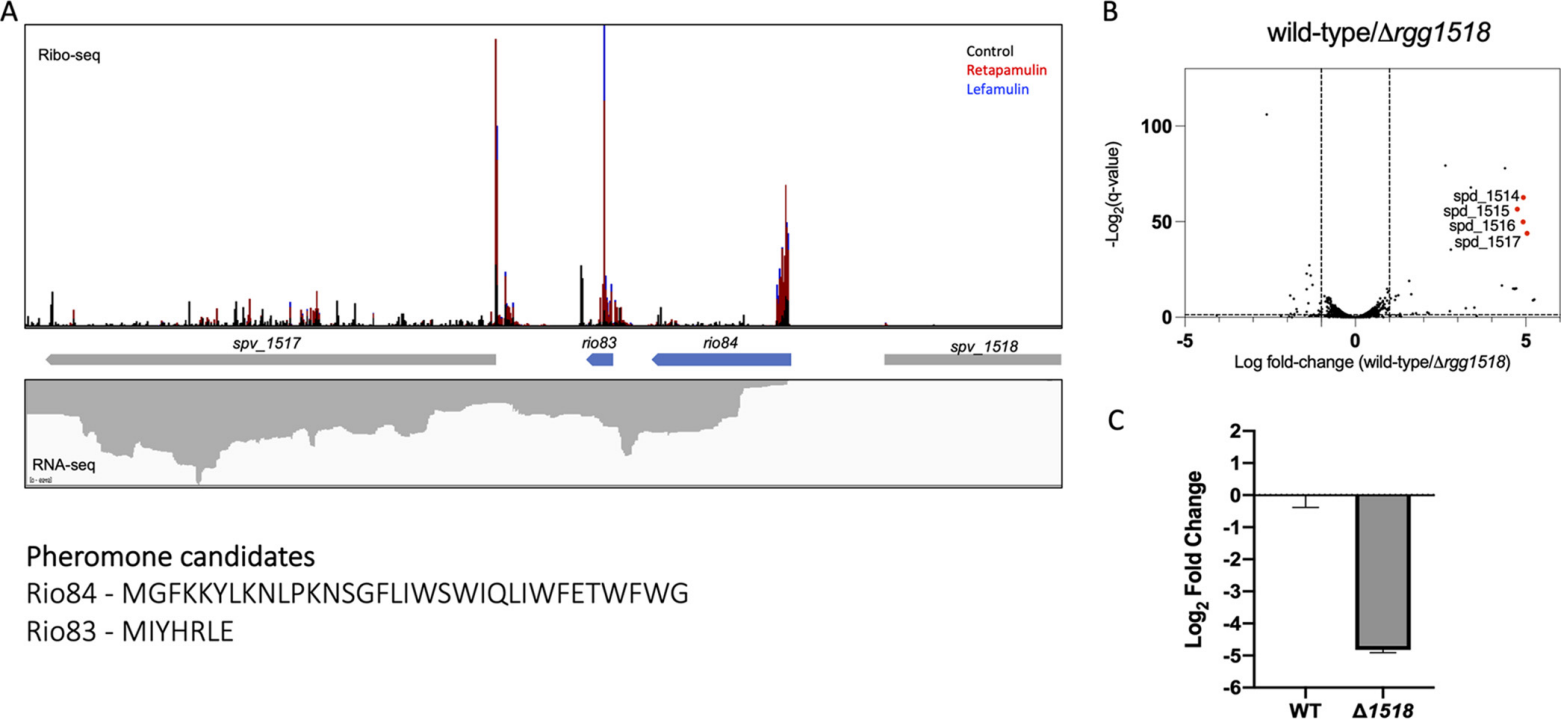
Identification of two novel sORFs found near the uncharacterized transcriptional regulator Rgg1518. (A) Ribosome footprint density profiles of *rio83* and *rio84* found near *spv_1518* (Rgg1518). Blue arrows represent sORFs identified by Ribo-RET, and gray arrows represent previously annotated ORFs. (B) Volcano plot of wild-type D39 versus Δ*rgg1518* transcript fold changes. Genes of interest with the highest fold change differences are indicated on the graph. (C) qRT-PCR validation of *spv_1517* expression in wild-type (WT) D39 versus the Δ*rgg1518* mutant.

Previous studies identified and characterized RRNPP transcriptional regulators in streptococci and demonstrated their importance in regulating genes associated with virulence, immunosuppression, lysozyme resistance, and competence (40–43). Ribo-RET detected the presence of two sORFs encoding polypeptides of 33 amino acids (*rio84*) and 7 amino acids (*rio83*) in length that are adjacent to an Rgg-like transcriptional regulator (*spv_1518*, referred to as *rgg1518* here) (Fig. 3A). The peptide encoded by *rio84* has characteristics resembling those of other streptococcal pheromones, such as a positively charged N terminus and a Trp-X-Trp (WXW) motif at the C terminus (44), leading us to hypothesize that *rio84* may encode the pheromone for Rgg1518 (Fig. 3A). To verify that Rgg1518 functions as a transcriptional regulator and to identify the genes under its regulation, transcriptome sequencing (RNA-seq) analysis was conducted to compare the gene expression of wild-type D39 to that of an isogenic deletion mutant, Δ*rgg1518* (Fig. 3B). The expression of the *spv_1513-1517* operon located adjacent to *rgg1518* and immediately downstream from *rio83* was substantially decreased in the deletion mutant, a trend that we verified by quantitative real-time PCR (qRT-PCR) (Fig. 3C). The operon of genes spv_1513 to spv_1517 (hereafter spv_1513-1517) encodes proteins predicted to comprise an ABC transporter of an unknown substrate(s), suggesting that Rgg1518 could be a regulator of nutrient acquisition. A previous report found that the *spv_1513-1517* operon was significantly upregulated when wild-type D39 bacteria were applied to A549 lung epithelial cells, suggesting a role during interactions with the host (25). We tested the impact that deleting the operon would have on adherence to or invasion of A549 cells but found no difference in attachment, internalization, or viability from the wild type, at least over short infection times (up to 4 h) (Fig. S5A and B). An independent recent report demonstrated that the presence of intact Rgg1518 is important for colonization of the murine nasopharynx by S. pneumoniae (23). To assess whether the *spv_1513-1517* operon is responsible for this phenotype, we coinfected CD1 mice with 10^5^ CFU of wild-type D39 and 10^5^ CFU of the Δ*spv_1513-1517* mutant in the nasopharynx and determined the bacterial burden in the nasal passage over a span of 7 days. The Δ*spv_1513-1517* mutant decreased over time in comparison with wild-type S. pneumoniae; however, the difference was not statistically significant, suggesting that the conditions in our experiment were not conducive to show whether this operon plays a critical role in colonizing the murine nasal passage (Fig. S5C).

10.1128/mbio.01247-22.5 FIG S5 The Rgg1518-regulated operon *spv_1513-1517* is not involved in adhesion, intracellular survival, or colonization of the murine nasopharynx. (A and B) A549 alveolar epithelial cells were infected with the Δ*cps* (positive control) and Δ*cps* Δ*spv_1513-1517* strains at an MOI of 1:100 to assess their role in adhesion (A) and intracellular survival (B). (C) CD1 mice were coinfected with 2 × 10^5^ CFU/20 μL of the wild-type D39 and Δ*spv_1513-1517* strains, and the bacterial burden in the nasopharynx was determined over a span of 7 days. Download FIG S5, TIF file, 1.7 MB. Copyright © 2022 Laczkovich et al. 2022 Laczkovich et al. https://creativecommons.org/licenses/by/4.0/ This content is distributed under the terms of the Creative Commons Attribution 4.0 International license.

**FIG 4:**
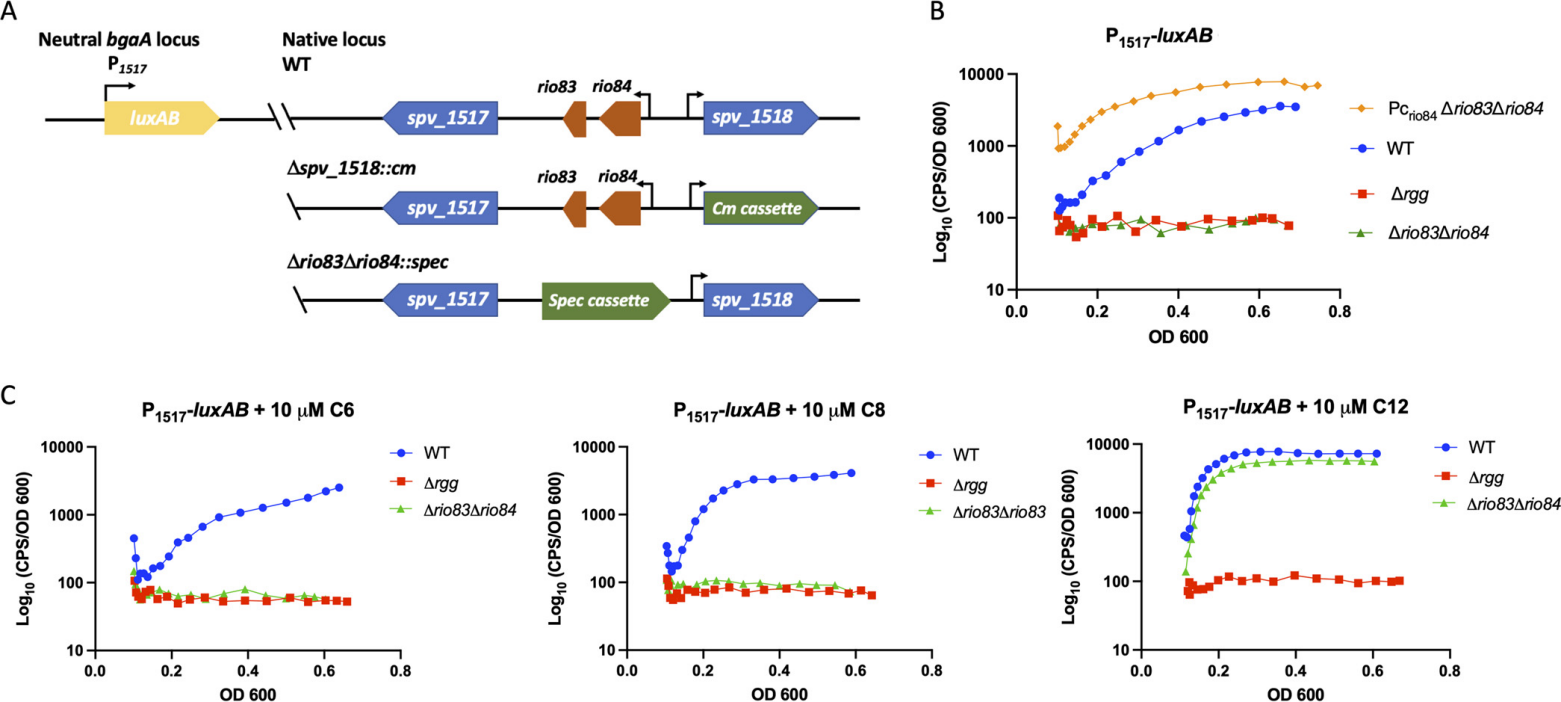
*rio84* encodes the signaling peptide for the Rgg1518 quorum sensing system. (A) Schematic of the luciferase reporter integrated into the *bgaA* locus of S. pneumoniae D39. The black arrows indicate the promoter. (B) P*_1517_* is induced when grown in CDM and upon the constitutive expression of *rio84* in the background of the Δ*rio83* Δ*rio84* strain. (C) Induction of P*_1517_* upon the addition of 10 μM synthetic C6, C8, and C12 Rio84 peptides. The data shown are representative of results from three independent experiments.

To assist in evaluating the potential contributions of Rgg1518, Rio83, and Rio84 to mediating cell-to-cell signaling, we constructed a luciferase-based transcription reporter using the promoter (P*_1517_*) identified by 5′ rapid amplification of cDNA ends (RACE) upstream of *rio84* (Fig. 4A). The promoter-reporter construct was placed into an unlinked, neutral location in the chromosome of isogenic strains with deletions of *rgg1518* or a combined deletion of its affiliated sORFs *rio83* and *rio84* (45). During growth in CDM, the wild-type reporter strain produced strong luminescence as the culture density increased, whereas the luminescence of the isogenic Δ*rgg1518* and Δ*rio83* Δ*rio84* mutants remained at low levels throughout the cultures’ growth (Fig. 4B; Fig. S6A). The expression of *rio84* from a constitutive promoter (P_c_-*rio84*) in the Δ*rio83* Δ*rio84* mutant background led to enhanced luciferase activity (Fig. 4B, yellow curve; Fig. S6A), indicating that the expression of *rio84* in *trans* was sufficient to complement the Δ*rio83* Δ*rio84* mutant. These results support a model in which *rio84* encodes a functional pheromone for Rgg1518, consistent with the results of a recent independent study (23). To identify the mature form of the pheromone, synthetic peptides of various lengths encompassing the C-terminal region of *rio84* (C6, C8, and C12) were added to cultures. While the 6- and 8-amino-acid-long peptides were unable to stimulate transcription from the P*_1517_* promoter, the C12 variant (IQLIWFETWFWG) efficiently induced the expression of P*_1517_* in the wild-type or Δ*rio83* Δ*rio84* strain but not in the Δ*rgg1518* strain (Fig. 4C; Fig. S6B). Thus, the active form of the *rio84* pheromone is likely confined within or is equivalent to this sequence.

**FIG 5:**
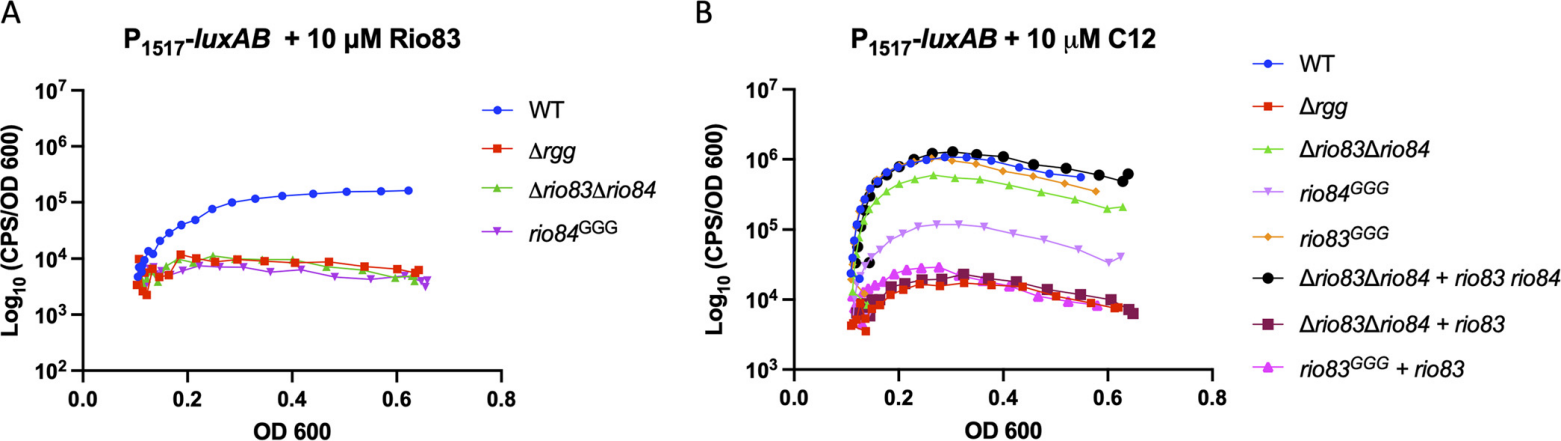
Expression of *rio83* in the absence of *rio84* represses luciferase activity. (A) P*_1517_* induction in the presence of 10 μM full-length synthetic Rio83. (B) P*_1517_* induction in different knockout strains in the presence of 10 μM synthetic C12. The data shown are representative of results from three independent experiments.

10.1128/mbio.01247-22.6 FIG S6 Biological replicates of luciferase assays. (A) Biological replicates of the luciferase assays depicted in Fig. 4B. (B) Biological replicates of the luciferase assays depicted in Fig. 4C. (C) Biological replicates of the luciferase assays in Fig. 5A. (D) Biological replicates of the luciferase assays in Fig. 5B. Download FIG S6, TIF file, 2.9 MB. Copyright © 2022 Laczkovich et al. 2022 Laczkovich et al. https://creativecommons.org/licenses/by/4.0/ This content is distributed under the terms of the Creative Commons Attribution 4.0 International license.

RNA-seq results indicated that *rio83* is downregulated in the absence of Rgg1518, suggesting that *rio83* might be involved in the regulation of Rgg1518-based QS regulation. The translation of the *rio83* sORF was validated by fusing it to *sfGFP* and was detected by Western blotting when cultures were treated with exogenous pheromone (Fig. 2C). The addition of the full-length synthetic *rio83* peptide to cultures did not alter luciferase activity (Fig. 5A; Fig. S6C). Intriguingly, the reporter activity in a Δ*rio84* mutant, grown in the presence of C12, did not reach the level of luciferase activity seen in the wild-type or Δ*rio83* Δ*rio84* strain (Fig. 5B; Fig. S6D). Furthermore, complementing the Δ*rio83* Δ*rio84* strain with *rio83* resulted in a complete loss of luminescence activity. These results suggest that *rio83* serves as a negative regulator. However, the extent of its impact on the control of the putative ABC transporter (*spv_1513-1517*) remains unclear.

The Ribo-RET data set also identified the known signaling peptide (*rio9*) for the Rgg0112 transcriptional regulator (44) as well as additional sORFs found downstream of Rgg0112 (*rio7*, *rio8*, and *rio10-15*) (Table 1), which appear to be part of the Rgg0112 regulon based on our RNA-seq data comparing wild-type D39 to an *rgg0112* mutant. Manual assessment of the Ribo-RET data set near other known Rgg-like transcriptional regulators identified sORFs that did not meet our initial search criteria (Table S3). *rio119*, found within the current annotation of *srf-06* and partially overlapping Rgg144, encodes the previously characterized pheromone for Rgg0144 (21, 22). Additional sORFs (*rio120*, *rio121*, and *rio122*) were identified within the same locus, downstream of the Rgg0144 pheromone and overlapping the transcriptional regulator on the opposite strand. To date, the roles that these additional sORFs may play in the QS systems are unknown.

10.1128/mbio.01247-22.10 TABLE S3 sORFs overlapping the gene on the opposite strand. Download Table S3, DOCX file, 0.1 MB. Copyright © 2022 Laczkovich et al. 2022 Laczkovich et al. https://creativecommons.org/licenses/by/4.0/ This content is distributed under the terms of the Creative Commons Attribution 4.0 International license.

### The sORF *rio3*, contained within the ncRNA *srf-02*, contributes to nasopharyngeal colonization.

**FIG 6:**
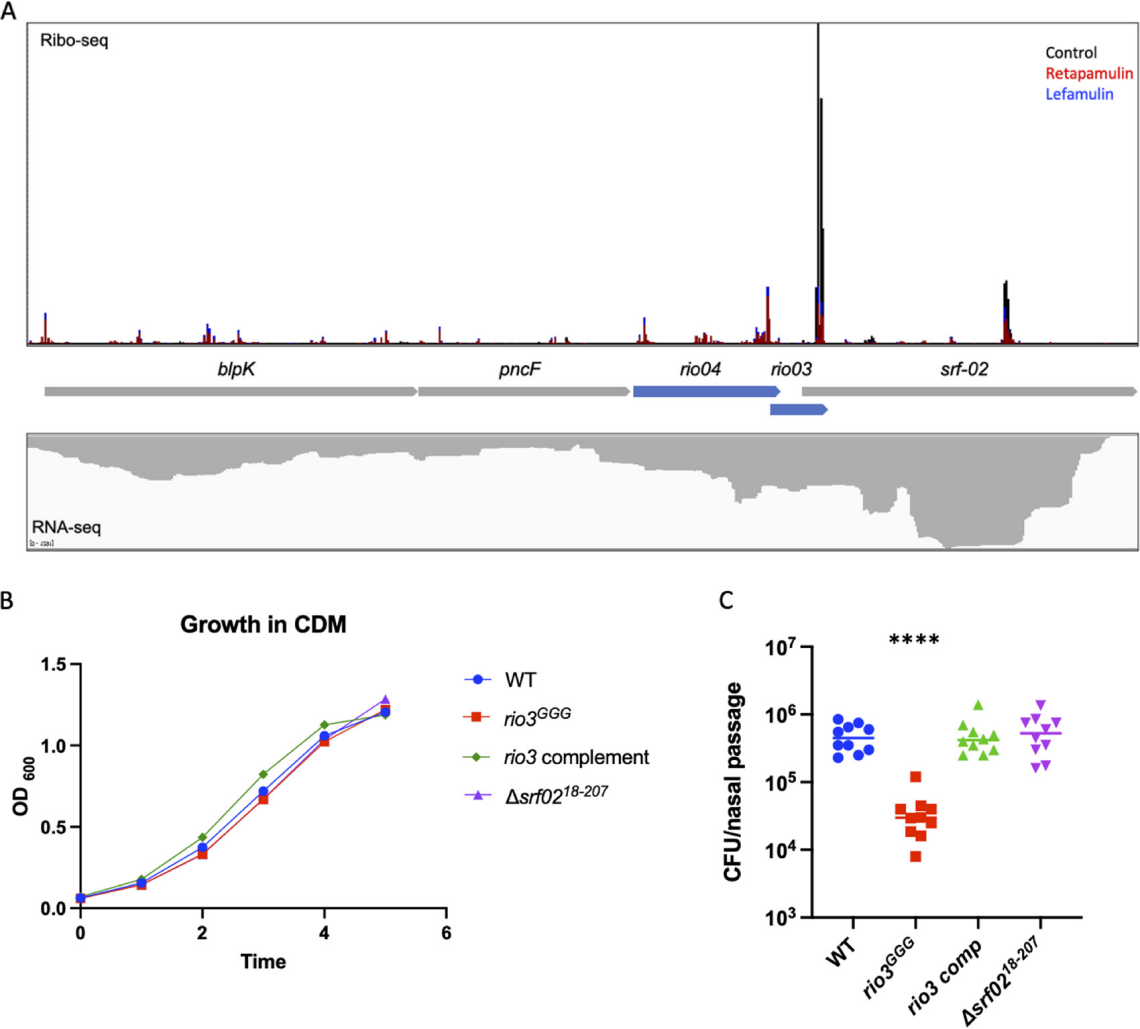
*rio3* is important for nasopharyngeal colonization in a pneumonia mouse model. (A) Ribosome footprint of the *blpK* operon. Arrows in blue represent sORFs identified by Ribo-RET, and arrows in gray represent ORFs annotated previously. (B) Growth curve of wild-type and mutant strains in CDM over a span of 6 h. (C) Six-week-old BALB/c mice were inoculated with 1 × 10^7^ CFU/25 μL of either the wild type, the *rio03^GGG^* mutant, the *rio03^GGG^* complemented strain, or the Δ*srf-02^18–207^* mutant. The nasal passages were collected at 24 h postinfection, homogenized, and plated to determine the bacterial burden. Statistical significance was determined using Kruskal-Wallis analysis. **** denotes a *P* value of <0.0001.

A previous TIS study identified a noncoding RNA, *F52*, in S. pneumoniae TIGR4 whose disruption negatively impacted the fitness of the pathogen in a mouse model of pneumonia (1). The S. pneumoniae reference strain D39 contains an ortholog of this ncRNA, which is referred to as *srf-02* (3). One of the sORFs identified and confirmed in our experiments (Fig. 2C), *rio3*, overlaps the annotated ncRNA *srf-02* (Fig. 6A). Given this overlap, we wondered if the fitness defect described in the TIS study might be attributable to a disruption of *rio3* rather than the ncRNA. To test this hypothesis, the start codon of *rio3* was mutated (ATG→GGG) to prevent the translation of the sORF. Separately, a deletion was generated (Δ*srf-02^18–207^*) that extended through the *srf-02* gene, which removed 188 3′-terminal nucleotides of the ncRNA while ensuring that the *rio3* sORF remained intact. Neither mutant strain displayed a growth defect compared to the wild type in culture (Fig. 6B). In order to assess whether *rio3* has an impact on nasopharyngeal colonization, 6-week-old BALB/c mice were infected intranasally with the wild type, the *rio03^GGG^* mutant, the Δ*srf-02^18–207^* mutant, or the *rio03^GGG^* complemented strain. The bacterial burdens in the nasal passage were enumerated at 24 h postinfection. Minimal differences were seen between the abilities of the wild-type, the Δ*srf-02^18–207^* mutant, and the *rio03^GGG^* complemented strains to colonize the nasopharynx; however, the *rio03^GGG^* mutant displayed a significant defect in colonizing the murine nasopharynx (Fig. 6C). These data indicate that the fitness defects attributed to *srf-02* on the basis of the TIS experiments are instead related to disruption of the sORF *rio3* identified in the S. pneumoniae genome by our Ribo-RET/Ribo-LEF approach.

## DISCUSSION

Ribosome profiling has been conducted and optimized extensively in E. coli; however, its application to other bacteria, including Gram-positive pathogens like S. pneumoniae, has seen limited reports (46–48). Here, we set out to identify actively translated unannotated sORFs using antibiotic-assisted ribosome profiling in S. pneumoniae D39, an approach that was successfully used to identify translation start sites in the E. coli genome (11, 29). We conducted profiling on samples without and with two translation inhibitors, retapamulin and lefamulin; identified 114 novel sORFs in the D39 genome; and confirmed that translation occurs for a subset of them. Although this is a considerable addition to the number of genes deserving future study in the S. pneumoniae genome, ribosome profiling provides only limited information regarding gene function. We drew upon genome context and published genomic studies to initiate a functional characterization of four sORFs: two associated with the Rgg1518 quorum sensing system, one attributed to colonization, and one serving as a leader peptide that governs that translation of peptide release factor. A total of 89% of the remaining sORFs were conserved in at least 2 genomes, and 42% were conserved in all 6 additional S. pneumoniae strains that we searched, representing diverse serotypes. Given the dynamic plasticity of the S. pneumoniae metagenome, the retention of sORFs among multiple genomes implies that they contribute to fitness, at least in some niches (Table 3). Identifying appropriate conditions under which an sORF contributes to fitness is not trivial, but having their identity known or proposed will stimulate hypothesis-driven mechanistic studies of bacterial processes in which sORFs are suspected to play a role.

**TABLE 3:** sORFs identified by Ribo-seq are conserved among other Streptococcus pneumoniae serotypes^*a*^

sORF length (nt)	sORF	Presence of sORF in strain					
		P1031 (serotype 1; GenBank accession no. CP000920)	TIGR4 (serotype 4; GenBank accession no. AE005672.3)	JJA (serotype 14; GenBank accession no. CP000919.1)	Hungary 19A-6 (serotype 19A; GenBank accession no. CP000936.1)	ATCC 700669 (serotype 23F; GenBank accession no. FM211187.1)	Taiwan 19F-14 (serotype 19F; GenBank accession no. CP000921.1)
32	*rio1*	X	X				X
19	*rio2*	X	X	X	X		X
11	***rio3***	X	X	X	X	X	X
28	*rio4*	X	X	X	X	X	X
37	*rio5*	X	X	X	X	X	X
28	*rio6*	X	X	X	X	X	X
25	*rio7*			X			X
9	***rio8***			X			
14	***rio9***			X			X
15	*rio10*		X				X
9	***rio11***		X				X
14	***rio12***		X		X	X	X
16	*rio13*	X			X		
21	*rio14*	X			X		X
7	***rio15***	X				X	X
11	***rio16***		X				X
20	*rio17*	X	X	X	X	X	X
13	*rio18*	X	X	X	X	X	X
28	*rio19*	X	X	X	X	X	X
26	*rio20*				X		X
13	***rio21***	X		X	X		
24	*rio22*						X
15	*rio23*	X	X	X	X	X	X
23	*rio24*	X	X	X	X	X	X
23	*rio25*	X	X	X	X	X	X
27	*rio26*	X	X	X	X	X	X
20	*rio27*	X	X	X	X	X	X
19	*rio28*	X	X	X	X	X	X
19	*rio29*	X	X	X	X	X	X
29	*rio30*		X	X	X		
13	*rio31*	X	X	X	X	X	X
9	***rio32***	X	X	X		X	
10	***rio33***	X	X	X	X	X	
17	*rio34* ^*b*^						
23	*rio35*	X	X	X	X	X	X
21	*rio36*	X	X	X	X	X	X
10	***rio37***	X	X		X	X	X
12	*rio38*			X	X		
24	*rio39*	X					
8	***rio40***	X	X		X	X	
21	*rio41*					X	
9	***rio42***	X				X	
14	*rio43*	X	X	X	X	X	X
13	*rio44*	X	X	X	X	X	X
16	*rio45*	X	X	X	X	X	X
8	***rio46***	X	X		X		
29	*rio47*	X	X	X	X	X	X
24	*rio48*	X	X	X	X	X	X
17	*rio49*		X	X	X	X	
30	*rio50*	X			X		X
19	*rio51*	X	X	X	X	X	X
27	*rio52*	X	X	X		X	X
11	***rio53***	X	X				
13	*rio54*	X	X	X	X	X	X
11	***rio55***				X		X
6	***rio56***	X	X		X		X
18	***rio57***	X		X		X	X
20	*rio58*	X	X	X	X	X	X
8	***rio59***		X				
16	*rio60*		X		X		X
9	***rio61***		X	X		X	
10	***rio62***	X		X		X	X
30	*rio63*	X	X	X	X	X	X
22	*rio64*	X	X	X	X	X	
21	*rio65*	X					
26	*rio66*	X	X	X	X	X	X
21	*rio67*	X	X	X	X	X	X
7	***rio68***	X	X			X	
12	*rio69*	X	X	X	X	X	X
17	*rio70*	X	X	X	X	X	X
23	*rio71*	X	X	X	X	X	X
10	***rio72*** ^*b*^						
12	*rio73*	X	X	X	X	X	X
23	*rio74*	X	X	X		X	X
5	***rio75***	X	X	X		X	
7	***rio76***	X	X	X		X	
31	*rio77*	X	X	X	X	X	X
42	*rio78*	X	X	X	X	X	X
24	*rio79*	X	X	X	X	X	X
14	*rio80*	X	X	X	X	X	X
8	***rio81***		X		X		
20	*rio82*	X	X	X	X	X	X
7	***rio83***			X	X	X	
33	*rio84*			X	X	X	
33	*rio85*			X			
16	*rio86*	X	X	X	X	X	X
14	*rio87*	X	X	X	X	X	X
37	*rio88*	X	X	X	X	X	X
10	***rio89*** ^*b*^						
35	*rio90*	X	X	X	X	X	X
21	*rio91*	X		X	X		X
7	***rio92***	X		X	X		X
21	***rio93***	X	X	X	X	X	X
9	***rio94***			X		X	X
32	*rio95*	X	X	X	X	X	X
9	***rio96***		X				X
6	***rio97***	X		X	X		X
8	***rio98***						X
10	***rio99***				X		
18	*rio100*	X			X		X
43	*rio101*	X	X	X	X	X	X
8	***rio102***	X		X			X
14	*rio103*	X	X	X	X	X	X
24	*rio104*	X	X	X	X	X	X
17	*rio105*	X		X		X	X
9	***rio106***		X	X	X	X	X
12	*rio107*	X	X	X	X	X	X
11	***rio108***	X	X	X		X	
9	***rio109***	X	X	X		X	
28	*rio110*	X	X	X	X	X	X
10	***rio111***			X			
30	*rio112*	X	X	X	X	X	X
9	***rio113***	X		X	X	X	X
5	***rio114***		X	X	X		X

a sORFs highlighted in boldface type were too short for tBLASTn analysis, so we assessed their conservation by looking for conserved nucleotide sequences.

b Nucleotide sequence not conserved in the 6 serotypes but found in other strains.

For instance, substantial effort has gone into identifying sORF-encoded pheromones of peptide-mediated QS systems (11, 22, 23, 29, 49–52). The number of putative pheromone receptors identified in genomes greatly outnumbers recognizable pheromone genes. Cognate pheromones for a majority of RRNPP proteins remain elusive since most receptor genes do not have an obvious pheromone-encoding sORF in their proximity; intergenic regions are typically replete with several theoretical sORFs, making it difficult to identify actual pheromone genes. In addition to the two sORFs associated with the Rgg1518 QS system, the Ribo-RET/LEF data set identified sORFs near previously characterized Rgg-mediated QS systems (Table 1), providing an empirical basis to test their role in QS systems. Unfortunately, translation profiling was still not powerful enough to predict pheromone sORFs for all RRNPP systems in S. pneumoniae, as the genes *rgg0999*, *rgg1786*, and *rgg1916* remain orphan receptors following our study. Transcription profiling (RNA-seq) indicated that the loci encoding these systems were silent under the conditions that we used to collect RNA and ribosomes. Thus, having conditions under which communication networks are universally active remains elusive and is a primary weakness of genome-wide expression studies.

Previous genomic studies conducted in S. pneumoniae D39, like those using transcriptional profiling tools and algorithms to annotate novel sRNAs (1, 2) and transposon insertion sequencing that correlates insertion mutants with fitness, were the primary sources of information for us to prioritize a deeper study of sORF function. Traditionally, sRNAs provide mechanisms of posttranscriptional regulation governing a variety of processes such as metabolism, the stress response, and virulence (53, 54). sRNAs are thought to be noncoding and function through base pair interactions with target mRNA molecules, either preventing or enhancing translation or influencing mRNA stability. The Ribo-RET and Ribo-LEF data sets identified sORFs within nine previously annotated sRNA loci, indicating that they either are protein-coding mRNAs or have a dual function as messengers and regulators. Our results argue that *rio3* is a protein-coding gene whose expression accounts for the *in vivo* fitness attribute first identified by TIS (1). It is possible that the *srf-02* RNA also plays a regulatory role in some fashion; however, we did not observe a phenotype supporting this possibility. Another ncRNA, *srf-21*, was found to contain the protein-coding gene *rio82*. Previous studies have shown that *srf-21* is regulated by the CiaRH two-component system known to regulate genes involved in competence, biofilm formation, antibiotic resistance, and stress tolerance (55, 56), suggesting a possible function of *rio82* in these processes.

An unexpected observation from the Ribo-RET/LEF data sets was the finding of a substantial number of genes for which ribosomes mapped to regions as far as 20 nt upstream of start codons (Fig. 1C); this was consistently observed among all 5 biological replicates (see Fig. S7 in the supplemental material). We have yet to determine whether these patterns are due to an unforeseen artifact of the modified techniques that we employed (i.e., elevated concentration of MgCl_2_ in the cell lysis buffer) or if they are attributable to a biological phenomenon. Since S. pneumoniae is an AT-rich organism, and the nuclease used to isolate the ribosome footprint (MNase) cleaves at A and U more efficiently than at G and C, we suspect that some mRNAs undergo aberrant digestion, leading to the incorrect mapping of the ribosome footprint. Our attempt to filter data based on footprint length improved the percentage of genes with aligned start sites, but a pattern of footprints in the 5′ untranslated region (UTR) remained albeit to a lesser extent. The use of a different nuclease, e.g., RNase I, or a combination of different nucleases could be a potential solution to mitigate the nuclease bias of AT-rich genomes in future ribosome profiling studies. However, we also cannot exclude that the presence of upstream ribosome footprints reflects an alternative mode of translation initiation in S. pneumoniae. The initiation of translation involves the recruitment of the ribosome to the ribosome binding site (RBS) in mRNA, aided sometimes by the recognition of a purine-rich SD sequence preceding the start codon (57–59). However, not every RBS contains conventional SD sequences, and a recent genome-wide study demonstrated that recognition of the SD motif is not crucial for translation initiation in E. coli (60). Additional factors might govern ribosome recruitment to the start codons of the ORFs. It is possible that the initiation of the translation of some genes in S. pneumoniae requires the loading of the ribosome upstream from the ORF, with the subsequent migration of the 70S initiation complex to the start codon.

Taken together, Ribo-RET is a powerful technique utilizing the initiation inhibitor retapamulin or lefamulin to reveal a genome-wide view of the translational landscape of S. pneumoniae D39. These data sets identify small proteins or microproteins whose contributions span a spectrum of activities that include cell-to-cell communication, host-microbe interactions, and physiological homeostasis.

## MATERIALS AND METHODS

### Bacterial strains, plasmids, and growth conditions.

All strains and plasmids used in this study are listed in Table S1 in the supplemental material. S. pneumoniae D39 was routinely grown on tryptic soy agar (TSA) supplemented with 5% sheep blood or cultured in Todd-Hewitt broth with 0.2% yeast (THY) and 0.5% Oxyrase (catalog number OB-0100; Oxyrase) or in a chemically defined medium (CDM) (50) supplemented with 1% glucose, 10% choline, and 0.5% Oxyrase at 37°C in an atmosphere of 5% CO_2_. When appropriate, chloramphenicol (4 μg/mL), spectinomycin (150 μg/mL), kanamycin (200 μg/mL), erythromycin (0.3 μg/mL), or neomycin (20 μg/mL) was added to S. pneumoniae D39 cultures.

10.1128/mbio.01247-22.8 TABLE S1 Strains and plasmids used in this study. Download Table S1, DOCX file, 0.02 MB. Copyright © 2022 Laczkovich et al. 2022 Laczkovich et al. https://creativecommons.org/licenses/by/4.0/ This content is distributed under the terms of the Creative Commons Attribution 4.0 International license.

### Transformation.

To generate competent S. pneumoniae D39 cells, wild-type D39 cells were grown in 7.5 mL THY supplemented with 0.013 N HCl and 0.05% glycine at 37°C in an atmosphere of 5% CO_2_ to an optical density at 600 nm (OD_600_) of 0.05 to 0.1. Cells were diluted into 1 mL THY to an OD_600_ of 0.03; supplemented with a solution containing 10 mM NaOH, 0.2% bovine serum albumin (BSA), 1 mM CaCl_2_, and 0.2 μg/mL competence-stimulating peptide 1 (CSP-1); and placed in a 37°C water bath for 14 min. Following incubation, ~850 ng of donor DNA was added, and cells were allowed to recover at 37°C with 5% CO_2_ for 1 h, followed by plating onto TSA plates supplemented with 5% sheep blood and the appropriate antibiotic.

### Construction of mutant strains.

All S. pneumoniae D39 deletion mutants, listed in Table S1, were generated by transforming competent S. pneumoniae D39 cells with linear DNA containing upstream and downstream sequences that facilitate homologous recombination and were generated by Gibson assembly of PCR amplicons using the primers listed in Table S2. All strains were confirmed by sequencing the locations of the chromosome containing the relevant alterations. Specific constructs are described further here. To delete *rgg1518* (strain IL20), a PCR-generated upstream flanking region (UFR) amplicon and a downstream flanking region (DFR) amplicon were joined with a chloramphenicol resistance cassette by Gibson assembly using NEBuilder HiFi DNA assembly master mix (New England BioLabs [NEB]). Strain IL40 (Δ*rio83* Δ*rio84*::*spec*) was constructed by Gibson assembly using a spectinomycin resistance cassette. Strain IL108 contains a deletion of the noncoding RNA *srf-02* (Δ*srf-02^18–207^*::*erm*) without disrupting the overlapping sORF *rio3*; the UFR encompasses the first 17 nucleotides of *srf-02*. To generate the missense point mutations in strains IL91 (*rio03^ATG-GGG^-spec*) and IL101 (*rio83^ATG-GGG^-spec*), special oligonucleotides were designed to replace the start codon ATG with the glycine codon GGG. To generate strain IL91, two DNA fragments were generated using primer pairs ILp355/ILp356 and ILp354/KTp043, and overlapping PCR was performed to generate a PCR amplicon with the start codon mutation in *rio3*, which was subsequently used as the template to amplify the UFR for the construct. To generate strain IL101, overlapping PCR was performed as described above, using primer pairs ILp170/ILp161 and ILp169/ILp166.

10.1128/mbio.01247-22.9 TABLE S2 Primers used for the construction of the strains used in this study. Download Table S2, DOCX file, 0.03 MB. Copyright © 2022 Laczkovich et al. 2022 Laczkovich et al. https://creativecommons.org/licenses/by/4.0/ This content is distributed under the terms of the Creative Commons Attribution 4.0 International license.

### Construction of chromosomal *luxAB* reporters.

To assess the expression levels of *spv_1517*, the intergenic region between *spv_1517* and *spv_1518* was amplified using a DNA template containing start codon mutations (GGG in place of ATG) in both *rio83* and *rio84*. To attain the DNA amplicon containing the missense mutations, overlapping PCR was performed using primer pairs ILp166/ILp167 and ILp168/ILp161 for *rio84* and primer pairs ILp170/ILp161 and ILp169/ILp166 for *rio83*. Overlapping PCR combined the two mutations on one DNA amplicon. The resulting linear piece of DNA was then used as a template to amplify the promoter region for *spv_1517* using primer pair ILp161/ILp166. Using Gibson assembly, the upstream region of the *bga* locus was fused to the promoter fragment and linked to luxAB of pJC156, followed by P_c_ and the kanamycin resistance cassette from CP1296 and flanked downstream by 2,000 bp of the *bgaA* gene. The resulting reporter construct was transformed into wild-type D39, IL20 (Δ*rgg1518*), IL40 (Δ*rio83* Δ*rio84*::*spec*), and IL101 (*rio83^ATG-GGG^-spec*).

To generate strain IL93 (Δ*rio83* Δ*rio84*::*spec bgaA*::P*_1517_^rio83-GGG^*^,^*^rio84-GGG^-luxAB-*P_c_*-kan-*P_c_*-rio84*), the luciferase reporter constitutively expressing *rio84* driven by the P_c_ promoter and genomic DNA from strain IL81 (*bgaA*::P*_1517_^rio83-GGG^*^,^*^rio84-GGG^-luxAB-*P_c_-*kan*) were used as the template to amplify the reporter construct using primer pair ILp387/ILp264, which was then linked to the constitutive promoter P_c_ and fused to *rio84* using Gibson assembly. This construct was transformed into wild-type D39 and IL40 (Δ*rio83* Δ*rio84*::*spec*).

### Restoration of mutations in *rio83* and *rio84* containing *luxAB* reporters.

Start codon mutations in *rio83* and *rio84* were restored by transforming strains IL40 (Δ*rio83* Δ*rio84*::*spec*) and IL101 (*rio83^ATG-GGG^-spec*) with DNA fragments containing wild-type sequences, generating strains IL52 (Δ*rio83* Δ*rio84*::*spec bgaA*::P_*1517*_^*rio83-ATG,rio84-ATG*^-*luxAB-*P_c_*-kan*), IL106 (Δ*rio83* Δ*rio84*::*spec bgaA*::P*_1517_^rio83-GGG^*^,^*^rio84-ATG^-luxAB-*P_c_-*kan*), and IL113 (*rio83^ATG-GGG^-spec bgaA*::P*_1517_^rio83-GGG^*^,^*^rio84-ATG^-luxAB-*P_c_*-kan*).

### Generation of chromosomal sfGFP-tagged constructs.

To generate the chromosomal superfolder GFP (*sfGFP*)-tagged constructs, we performed transformations using linear DNA amplicons as described above. Each construct fused *sfGFP* in frame in front of the stop codon, followed by a spectinomycin resistance cassette, and was flanked by UFR and DFR homologous sequences. Strain IL75 (D39 *rio03^ATG-GGG^-sfGFP*) was constructed using strain IL91 (*rio03^ATG-GGG^-spec*) as a template to amplify the missense mutation with primer pair ILp323/ILp324.

### SDS-PAGE and Western blotting for sfGFP-tagged sORFs.

The *sfGFP*-tagged strains were grown in 10 mL CDM to an OD_600_ of 0.4, and cells were collected at 4,000 × *g* for 10 min. Cell pellets were resuspended in 250 μL loading buffer (0.0625 M Tris [pH 8], 2% SDS, 10% glycerol, 5% 2-mercaptoethanol, 50 mg bromophenol blue) and lysed using a BioSpec bead beater for 10 min at maximum speed. Gel loading volumes of each sample were normalized by culture OD readings and resolved on a 12% SDS-PAGE gel at 150 V for 1.5 h. Gels were blotted onto 0.2-μm polyvinylidene difluoride (PVDF) membranes at 350 mA for 1.5 h, and the membranes were blocked overnight at 4°C with rocking in Tris-buffered saline plus 0.1% Tween (TBST) containing 5% BSA. Membranes were subsequently incubated for 1 h at room temperature, with rocking, with anti-sfGFP antibody (catalog number AE011; ABclonal) at a dilution of 1:3,000 in TBST plus 5% BSA. The membranes were then washed three times in TBST, followed by the addition of goat anti-rabbit IgG(H+L) (Thermo Fisher) at a dilution of 1:80,000 in TBST plus 5% BSA for 1 h with rocking at room temperature. The membranes were then washed three times, and sfGFP-tagged proteins were detected using the SuperSignal West Femto maximum-sensitivity substrate (catalog number 34094; Thermo Fisher). To prepare the working solution, equal volumes of the stable peroxide solution and the luminol-enhancer solution were mixed and incubated with the blot for 5 min, followed by exposure on a ProteinSimple FluorChem imaging system.

### Synthesis of pheromone peptides.

Synthetic peptides were purchased from ABclonal. All peptides were reconstituted in dimethyl sulfoxide (DMSO) at a final concentration of 10 mM and stored at −80°C. Peptide purity ranged from 50 to 80%.

### MIC assay.

Dilutions of the antibiotics retapamulin and lefamulin were prepared in CDM and loaded into a 96-well microtiter plate. D39 Δ*cps* was grown in CDM to an OD_600_ of 0.5 and diluted 10-fold to an OD_600_ of 0.05 into the antibiotic-containing medium. Plates were incubated at 37°C in a microplate reader (Synergy 2; BioTek), and the OD was measured every 15 min over a span of 10 h.

### Metabolic labeling.

Inhibition of translation by retapamulin and lefamulin was determined using metabolic labeling. All manipulations were performed at 37°C. D39 Δ*cps* was inoculated from a starter culture (OD_600_ of 1) into 6 mL and grown in CDM lacking methionine and containing 0.5% Oxyrase to an OD_600_ of 0.5 at 37°C with 5% CO_2_. Cells were diluted 10-fold into CDM without methionine and containing 0.5% Oxyrase and grown until the culture density reached an OD_600_ of ~0.2, and three 350-μL aliquots were transferred to microcentrifuge tubes (two drug conditions and one control group). Retapamulin and lefamulin were individually added to Eppendorf tubes at a final concentration of 100× MIC. Prior to and immediately following the addition of antibiotics (0, 1, 2.5, 5, and 15 min), 28 μL of the culture was added to microcentrifuge tubes containing 0.3 μCi [^35^S]l-methionine (specific activity of 1,175 Ci/mmol; MP Biomedicals) in 2 μL of CDM. After a 1-min incubation, 25 μL of the mixture was spotted onto Whatman 3MM paper discs prewetted with 7% trichloroacetic acid (TCA). The discs were boiled twice in 7% TCA for 5 min, soaked in 100% acetone for 2 min, and then air dried prior to being placed into a 5-mL scintillation cocktail and being read using a scintillation counter.

### Ribosome profiling.

Ribosome profiling was conducted as previously described, with the following modifications (29, 61). D39 Δ*cps* cells were grown to an OD_600_ of 0.4 in 100 mL CDM supplemented with 0.5% Oxyrase at 37°C in an atmosphere of 5% CO_2_. Retapamulin or lefamulin was added to individual 100-mL cultures at final concentrations of 100× MIC for 2.5 min. No antibiotic was added to the untreated control group. After 2.5 min, bacteria were harvested by centrifugation at 6,300 × *g* at 37°C for 4 min and flash-frozen in liquid nitrogen. Cells were cryo-lysed in 650 μL lysis buffer (20 mM Tris-HCl [pH 8.0], 50 mM MgCl_2_, 100 mM NH_4_Cl,5 mM CaCl_2_) supplemented with 65 U RNase-free DNase I (catalog number 04716728001; Roche), 208 U SUPERase In RNase inhibitor (catalog number AM2694; Invitrogen), and 3 mM Guanosine 5′ –[b,g-imido] triphosphate trisodium salt hydrate (GMPPNP; catalog number G0635; Sigma-Aldrich). Pulverized cells were thawed at 30°C and spun at 20,000 × *g* for 10 min at 4°C to pellet insoluble debris. Clarified lysates were subjected to treatment with 450 U MNase (catalog number 10107921001; Roche), 120 U SUPERase In RNase inhibitor was added to the clarified lysates, and the reaction mixtures were incubated for 1 h at 25°C with shaking. The reaction mixtures were quenched with 5 mM EGTA, and the 70S monosome peak was isolated by sucrose gradient centrifugation (10 to 40% sucrose gradient) for 2 h 45 min at 39,000 *× g*. RNA was isolated by acid-phenol extraction and run on a 15% Tris-borate-EDTA (TBE)-urea polyacrylamide gel. RNA fragments ranging from 20 nucleotides to 38 nucleotides were excised, eluted, and used for library preparation as previously described, which included the addition of barcodes for multiplexing (31).

### Computational analysis of ribosome profiling data.

The ribosome footprint reads were analyzed as described previously (61). In brief, samples were demultiplexed, linker barcodes were removed, and 5 nucleotides from the 3′ end and 2 nucleotides from the 5′ end were removed as they were included in the library design (29, 31). The reads were aligned to the S. pneumoniae D39V (GenBank accession number CP027540.1) reference genome by Bowtie2 (v2.2.9) after discarding reads mapping to known tRNAs and rRNAs. Read lengths ranging from 28 to 34 nucleotides were included for the analysis; the first nucleotide of the P-site codon was assigned 15 nucleotides from the 3′ end of the read, as previously suggested (11).

Novel sORFs found within intergenic regions were identified based on the following criteria: a Ribo-RET peak of at least 1 sequence read per million (rpm) that mapped within 10 nucleotides of a theoretical sORF starting with AUG, GUG, CUG, or UUG and whose respective full-length sORF did not overlap an annotated gene. In some instances, multiple start codons were identified in the 10-nucleotide window; therefore, a manual approach was used to inspect each candidate relative to the Ribo-RET peak. The list of sORFs identified can be found in Table 1. The code used to analyze the data set can be found at https://github.com/ilaczk2/D39_ribosome_profile_MS.

### Metagene analysis.

Metagene analyses, to evaluate the positions of ribosomes at annotated genes with respect to the 5′ (start) and 3′ (stop) ends of genes, were performed according to a previously described protocol (62). Genes included in the analysis satisfied two criteria: a length of at least 200 nt and a read density of at least 0.005 rpm per nucleotide in all 5 samples (2 retapamulin, 1 lefamulin, and 2 controls). Coverage at each nucleotide position within a gene was normalized to the coverage density of the entire gene plus 50 nt of the flanking up- and downstream regions. The mean of these values was calculated and plotted for the windows around the start and stop codons.

### Luciferase assay.

Strains of interest were inoculated from flash-frozen starter cultures in CDM plus 0.5% Oxyrase at 37°C in an atmosphere of 5% CO_2_ and reached exponential growth to an OD of ~0.5. Strains were then diluted 10-fold in CDM in a total volume of 150 μL in a 96-well white/clear-bottom plate (Sigma). When relevant, synthetic peptides were added to the wells at a final concentration of 10 μM. Dosing assays determined 10 μM to be the optimal concentration to induce the system. Decyl aldehyde (Sigma) was added to the spaces between the wells at a final concentration of 1% in mineral oil. The plate was covered and placed into the microplate reader (Synergy 2; BioTek) at 37°C with intermittent shaking. The luminescence (counts per second [CPS]) and optical density (OD_600_) were measured every 15 min over a span of 10 h. Relative light units (RLU) were calculated by normalizing the CPS to the OD_600_. Each assay was conducted in technical triplicates, and each figure shows results representative of data from at least three independent experiments (Fig. S6).

### Cell adhesion assay.

A549 lung epithelial cells (ATCC) were routinely cultured in Dulbecco’s modified Eagle’s medium (DMEM) plus 10% fetal bovine serum (FBS) without antibiotics at 37°C in an atmosphere of 5% CO_2_. For the adhesion assay, A549 cells were seeded into a 24-well plate at 2 × 10^5^ cells/well. Following incubation overnight, each well was washed once with 1× phosphate-buffered saline (PBS). D39 Δ*cps* and D39 Δ*cps* Δ*spv_1513-1517*::*spec* were grown in CDM plus 10% choline and 0.5% Oxyrase to an OD_600_ of 0.5, washed in DMEM, and added to the cells at a multiplicity of infection (MOI) of 100:1 for 1 h at 37°C with 5% CO_2_. Following incubation, cells were gently washed three times with 1× PBS to remove unbound bacteria, treated with 0.025% trypsin for 6 min at 37°C with 5% CO_2_ to detach the cells, lysed with 0.1% saponin, and plated onto blood agar plates to determine bacterial CFU.

### Intracellular survival assay.

The initial steps for the intracellular survival assay were the same as the ones described above for the adhesion assay. To differentiate between internalized and external bacteria, epithelial cells were treated with 100 μg/mL gentamicin for 1, 3, and 4 h to kill the external bacteria. Following antibiotic treatment, the supernatant was aspirated, and cells were gently washed three times with 1× PBS. Cells were removed from the wells by the addition of 0.025% trypsin for 6 min and lysed with 0.1% saponin. The suspension was serially diluted and plated onto sheep blood agar plates to determine the bacterial burden.

### Mouse experiment.

Mice were housed at a biosafety level 2 facility and anesthetized with inhaled isoflurane (3%) when necessary. As shown in Fig. 6C, 6-week-old BALB/c mice were intranasally inoculated with wild-type D39, IL91 (*rio03^ATG-GGG^-spec*), IL127 (*rio03^ATG-GGG^*;*rio03^GGG-ATG^-kan*), and IL108 (Δs*rf-02^18–207^*::*erm*) at a dose of 1 × 10^7^ CFU/25 μL. A minimum of 10 mice were used for each bacterial inoculation. Mice were sacrificed 24 h after inoculation using carbon dioxide inhalation followed by cervical dislocation. The nasal passage of each mouse was isolated, homogenized in 500 μL 1× PBS, and plated onto blood agar plates to determine the bacterial CFU burden. As shown in Fig. S6C, 30 6-week-old CD1 mice were intranasally inoculated with bacterial suspensions containing 1:1 mixtures of wild-type D39 and IL97 (Δ*spv_1513-1517*::*spec*) at a dose of 2 × 10^5^ CFU/20 μL. Ten mice were sacrificed either 24 h, 72 h, or 168 h after inoculation, followed by nasal passage isolation and plating onto blood agar plates to determine bacterial CFU.

### RNA isolation and RNA sequencing.

The wild-type D39 and IL20 (Δ*rgg1518*) strains were cultured in CDM supplemented with 10% choline plus 0.5% Oxyrase and grown to an OD_600_ of 0.4 at 37°C with 5% CO_2_. Three independent cultures of each strain were prepared. Cultures were harvested by centrifugation, supernatants were discarded, and cell pellets were suspended in 1 mL RNAlater (Ambion) and incubated at room temperature for 10 min. Following incubation, samples were centrifuged at 14,000 × *g* for 1 min, supernatants were discarded, and cell pellets were stored at −80°C. Total RNAs from wild-type D39, IL20 (Δ*rgg1518*), and the Ribo-seq samples (retapamulin treated, lefamulin treated, and untreated) were extracted using the Ambion RiboPure RNA purification bacterial kit according to the manufacturer’s instructions and as previously described (63). Following the successful extraction of RNA, the Genome Research Core at the University of Illinois at Chicago (UIC) assessed RNA quality and quantity using the Tapestation 2200 system (Agilent), prepared the cDNA libraries, and processed samples on an Illumina HiSeq 4000 platform with 100-bp single reads. The raw sequencing data were analyzed by the Research Informatics Core at UIC.

### Preparation of cDNA for qRT-PCR experiments.

cDNA was prepared from RNA using the Superscript III first-strand synthesis system (Thermo Fisher) according to the manufacturer’s instructions and as previously described (63). Total cDNA was diluted 1:10, and reaction mixtures were prepared using 1× Fast SYBR green master mix with the gene-specific primers listed in Table S2. qRT-PCR was performed using the CFX Connect real-time PCR detection system (Bio-Rad). All samples were run in biological and technical triplicates, and relative gene expression was determined using the 2^−ΔΔ^*^CT^* method.

### 5′ RACE.

5′ RACE was conducted as previously described (63). Total RNA was isolated as detailed in the section on RNA isolation and RNA sequencing above. cDNA synthesis of the *spv_1517* transcript and template switching were performed using the NEB template-switching RT enzyme mix with primers specific for the *spv_1517* operon (ILp151) and the template-switching oligonucleotide (TSO) (BRp311). The 5′ end of the *spv_1517* transcript was amplified using IL151 primers and the TSO-specific primer BRp312 using the Q5 high-fidelity enzyme. The resulting PCR product was sequenced by Sanger sequencing.

### sORF conservation analysis.

sORF conservation was assessed as previously described (64). tBLASTn analysis was used to assess sORF conservation in six clinically relevant S. pneumoniae serotypes (1, 4, 14, 19A, 23F, and 19F). The amino acid sequence of each sORF was submitted to tBLASTn analysis. The following parameters were modified: the maximum number of target sequences was 250, the expected threshold was set to 100, and no low-complexity filter was used. The search was refined by selecting only sORFs that had 100% query coverage and ≥70% sequence identity. The sORFs highlighted in boldface type in Table 3 were too short for tBLASTn analysis; therefore, BLASTn was used to assess their conservation using the same parameters as the ones described above.

### Data availability.

All raw ribosome profiling reads, RNA sequencing reads, and annotation files are available in the NCBI GEO database (https://www.ncbi.nlm.nih.gov/bioproject/857299; Accession #PRJNA857299). Analysis scripts are available on GitHub (https://github.com/ilaczk2/D39_ribosome_profile_MS).
