# Supplementary material for: Discovery of Unannotated Small Open Reading Frames in Streptococcus pneumoniae D39 Involved in Quorum Sensing and Virulence Using Ribosome Profiling
Source: mBio. 2022 Jul 19;13(4):e01247-22. doi: 10.1128/mbio.01247-22 (PMC9426450; doi:10.1128/mbio.01247-22)
Supplement: TABLE S1 [file mbio.01247-22-s0009.docx]

**S1 Table. Strains and plasmids used in this study**

| **Strain** | **Description** | **Antibiotic Resistance** | **Reference** |
| --- | --- | --- | --- |
| IL01 | *Streptococcus pneumoniae* D39 wild-type, from M. E. Winkler | NA | ^1^ |
| IL24 | D39 Δ*cps*, from M. E. Winkler | marker less | ^2^ |
| CP1296 | R6 *cbp3*::kan-rpsL^+^ | Kan^R^ | ^3^ |
| IL20 | D39 Δ*rgg1518* | Cm^R^ | This study |
| IL110 | D39 Δ*cps* Δ*spv_1513-1517::spec* | Spec^R^ | This study |
| IL97 | D39 Δ*spv_1513-1517::spec* | Spec^R^ | This study |
| IL40 | D39 Δ*rio83*Δ*rio84::spec* | Spec^R^ | This study |
| IL108 | D39 Δ*srf02^18-207^::erm* | Erm^R^ | This study |
| IL92 | D39 *rio03^ATG-GGG^-spec* | Spec^R^ | This study |
| IL127 | D39 *rio03^ATG-GGG^;rio03^GGG-ATG^-kan* | Kan^R^ | This study |
| IL101 | D39 *rio83^ATG-GGG^-spec* | Spec^R^ | This study |
| IL48 | D39 *rio49-sfGFP* | Spec^R^ | This study |
| IL47 | D39 *rio85-sfGFP* | Spec^R^ | This study |
| IL61 | D39 *rio106-sfGFP* | Spec^R^ | This study |
| IL63 | D39 *rio48-sfGFP* | Spec^R^ | This study |
| IL68 | D39 *rio01-sfGFP* | Spec^R^ | This study |
| IL59 | D39 *rio83-sfGFP* | Spec^R^ | This study |
| IL64 | D39 *rio97-sfGFP* | Spec^R^ | This study |
| IL66 | D39 *rio03-sfGFP* | Spec^R^ | This study |
| IL45 | D39 *rio56-sfGFP* | Spec^R^ | This study |
| IL46 | D39 *rio82-sfGFP* | Spec^R^ | This study |
| IL75 | D39 *rio03^ATG-GGG^-sfGFP* | Spec^R^ | This study |
| IL81 | D39 wild-type *bgaA::* P_1517_*^rio83^*^-GGG,^ *^rio84^*^-GGG^-*luxAB-P_c_-kan* | Kan^R^ | This study |
| IL72 | D39 Δ*rgg1518::cm bgaA::* P_1517_*^rio83^*^-GGG,^ *^rio84^*^-GGG^-*luxAB-P_c_-kan* | Kan^R^, Cm^R^ | This study |
| IL83 | D39 Δ*rio83*Δ*rio84::spec bgaA::* P_1517_*^rio83^*^-GGG,^ *^rio84^*^-GGG^-*luxAB-P_c_-kan* | Kan^R^, Spec^R^ | This study |
| IL95 | D39 wild-type *bgaA::* P_1517_*^rio83^*^-GGG,^ *^rio84^*^-GGG^-*luxAB-P_c_-kan-P_c_-rio84* | Kan^R^, Spec^R^ | This study |
| IL93 | D39 Δ*rio83*Δ*rio84::spec bgaA::* P_1517_*^rio83^*^-GGG,^ *^rio84^*^-GGG^-*luxAB-P_c_-kan-P_c_-rio84* | Kan^R^, Spec^R^ | This study |
| IL103 | D39 *rio84^ATG-GGG^-spec bgaA::* P_1517_*^rio83^*^-GGG,^ *^rio84^*^-GGG^-*luxAB-P_c_-kan* | Kan^R^, Spec^R^ | This study |
| IL115 | D39 *rio83^ATG-GGG^-spec bgaA::* P_1517_*^rio83^*^-GGG,^ *^rio84^*^-GGG^-*luxAB-P_c_-kan* | Kan^R^, Spec^R^ | This study |
| IL52 | D39 Δrio83Δ*rio84::spec bgaA::* P_1517_*^rio83^*^-ATG,^ *^rio84^*^-ATG^-*luxAB-P_c_-kan* | Kan^R^, Spec^R^ | This study |
| IL106 | D39 Δ*rio83*Δ*rio84::spec bgaA::* P_1517_*^rio83^*^-GGG,^ *^rio84^*^-ATG^-*luxAB-P_c_-kan* | Kan^R^, Spec^R^ | This study |
| IL113 | D39 *rio83^ATG-GGG^ -spec bgaA::* P_1517_*^rio83^*^-GGG,^ *^rio84^*^-ATG^-*luxAB-P_c_-kan* | Kan^R^, Spec^R^ | This study |
|  | | | |
|  | | | |
| **Plasmids and PCR templates** | | | |
| pY71-sfGFP | Source of *sfGFP* tag | Kan^R^ | ^4^ |
| pLZ12Spec | Shuttle vector encoding spectinomycin resistance cassette | Spec^R^ | ^5^ |
| pEVP3 | Template plasmid for chloramphenicol cassette | Cm^R^ | ^6^ |
| pJC156 | Vector encoding *luxAB* genes | Erm^R^ | ^7^ |
| pFED760 | Template plasmid for erythromycin cassette | Erm^R^ | ^7^ |
| P*c* | TAGGATCCGTTTGATTTTTAATGGATAATGTGATATAATCTTTAAATACTGTAGAAAAGAGGAAGGAAATAATAA | Constitutive promoter sequence | ^6^ |

**References**

(1) Lanie JA, Ng W, Kazmierczak KM, Andrzejewski TM, Davidsen TM, Wayne KJ, et al. Genome sequence of Avery's virulent serotype 2 strain D39 of Streptococcus pneumoniae and comparison with that of unencapsulated laboratory strain R6. J Bacteriol 2007 -01;189(1):38-51.

(2) Magee AD, Yother J. Requirement for Capsule in Colonization by Streptococcus pneumoniae. Infect Immun 2001 -6;69(6):3755-3761.

(3) Charpentier E, Anton AI, Barry P, Alfonso B, Fang Y, Novick RP. Novel cassette-based shuttle vector system for gram-positive bacteria. Appl Environ Microbiol 2004 -10;70(10):6076-6085

(4) Bundy BC, Swartz JR. Site-specific incorporation of p-propargyloxyphenylalanine in a cell-free environment for direct protein-protein click conjugation. Bioconjug Chem 2010 -02-17;21(2):255-263.

(5) Husmann LK, Scott JR, Lindahl G, Stenberg L. Expression of the Arp protein, a member of the M protein family, is not sufficient to inhibit phagocytosis of Streptococcus pyogenes. Infect Immun 1995 -01;63(1):345-348.

(6) Claverys JP, Dintilhac A, Pestova EV, Martin B, Morrison DA. Construction and evaluation of new drug-resistance cassettes for gene disruption mutagenesis in Streptococcus pneumoniae, using an ami test platform. Gene 1995 -10-16;164(1):123-128.

(7) Mashburn-Warren L, Morrison DA, Federle MJ. A novel double-tryptophan peptide pheromone controls competence in Streptococcus spp. via an Rgg regulator. Mol Microbiol 2010 Nov;78(3):589-606.
