# Supplementary material for: Discovery of Unannotated Small Open Reading Frames in Streptococcus pneumoniae D39 Involved in Quorum Sensing and Virulence Using Ribosome Profiling
Source: mBio. 2022 Jul 19;13(4):e01247-22. doi: 10.1128/mbio.01247-22 (PMC9426450; doi:10.1128/mbio.01247-22)
Supplement: TABLE S2 [file mbio.01247-22-s0010.docx]

**S2 Table. Primers used for the construction of the strains used in this study**

| **Primer** | **Sequence (5’ to 3’)** | **Template** | **Amplicon** | **Comments** |
| --- | --- | --- | --- | --- |
| Construction of IL20 (D39 Δ*rgg1518*) | | | | |
| ILp015 | ATCAGATAAGACAGTAGTAAGTACAAC | D39 | UFR |  |
| ILp058 | caaacaaattttcatcaagcATTGTTCCTCCTAGAAAAATG | D39 | UFR |  |
| JC100 | gcttgatgaaaatttgtttgattt | pEVP3 | CM |  |
| JC101 | gatgggttccgaggctc | pEVP3 | CM |  |
| ILp059 | gttgagcctcggaacccatcTTTGTAATAACTCTCATCATTTTATTATAG | D39 | DFR |  |
| ILp018 | TATCGGTACAGGGGATGTAC | D39 | DFR |  |
|  | | | | |
| Construction of IL110 (D39 Δ*cps* Δ*spv_1513-1517::spec*) | | | | |
| ILp406 | TGATGACATCGTAACGTTGAG | D39 | UFR |  |
| ILp407 | caatttttttAGTATCATAAAAGTCAGAGAGGTTAG | D39 | UFR |  |
| ILp408 | tttatgatactAAAAAAATTGAAAAAAGTGTTTCCAC | pLZ12spec | spec |  |
| ILp409 | aacagaaaaaTCGATTTTCGTTCGTGAATAC | pLZ12spec | spec |  |
| ILp410 | cgaaaatcgaTTTTTCTGTTTCCTTTCATTTTTTATTC | D39 | DFR |  |
| ILp411 | TTTCTTTTTATTCTGCTAGCTATTG | D39 | DFR |  |
|  | | | | |
| Construction of IL40 (D39 Δ*rio83*Δ*rio84::spec*) | | | | |
| ILp161 | AGTTAAGAGAGGAATCTTCATAAATTATAC | D39 | UFR |  |
| ILp162 | caatttttttGTCTATATTTAAAATAGAAGTTATAAGGAATC | D39 | UFR |  |
| ILp163 | taaatatagacAAAAAAATTGAAAAAAGTGTTTCCAC | pLZ12spec | spec |  |
| ILp164 | ggaaaaagtcATGGAAATGAAAGATCATATCATATATAATC | pLZ12spec | spec |  |
| ILp165 | tcatttccatGACTTTTTCCCTCCTTTTTGATATAATAATAC | D39 | DFR |  |
| ILp166 | AAGACGTGGTTGTTTTTGAATC | D39 | DFR |  |
|  | | | | |
| Construction of IL108 (D39 Δ*srf02^18-207^::erm*) | | | | |
| ILp323 | CCAAGGCTGTCAAAGATG | D39 | UFR |  |
| ILp451 | gattatatcacattatccattaaaaatcaaacggatcctatcagaatagataacaccgttctaaattac | P_c_ | P_c_ | Primer contains P_c_ sequence |
| BR7 | Tgatataatctttaaatactgtagaaaagaggaaggaaataataaatgaacaaaaatataaaatattctcaaaacttttt | pFED760 | Erm | Primer contains P_c_ sequence and part of erm cassette |
| ILp426 | aaagagccgaTTATTTCCTCCCGTTAAATAATAGATAAC | pFED760 | Erm |  |
| ILp427 | gaggaaataaTCGGCTCTTTGTCAACTG | D39 | DFR |  |
| ILp202 | GGAGCAATTTCCCTTCCTTAAG | D39 | DFR |  |
|  | | | | |
| Construction of IL91 (D39 *rio03^ATG-GGG^-spec*) | | | | |
| ILp355 | gaatagataacaccgttctaaattacgattcccagttactccttttttgactcgttactataa | D39 | srf02^GGG^ | PCR stitching IL356/KT043 |
| ILp356 | atgagaaatatcggacaagc | D39 | srf02^GGG^ |  |
| ILp354 | ttatagtaacgagtcaaaaaaggagtaactgggaatcgtaatttagaacggtgttatctattc | D39 | srf02^GGG^ |  |
| KT043 | atggaacaattacattttatcacaaaattac | D39 | srf02^GGG^ |  |
| ILp357 | cgaaaatcgaACCCACTACAGTTGACAAAG | srf02^GGG^ | UFR | Combined with IL356 |
| ILp358 | tgtagtgggtTCGATTTTCGTTCGTGAATAC | pLZ12spec | Spec |  |
| ILp359 | gctttttttcaAAAAAAATTGAAAAAAGTGTTTCCAC | pLZ12spec | Spec |  |
| ILp360 | caatttttttTGAAAAAAAGCTAAGCTCGAG | D39 | DFR |  |
| ILp202 | GGAGCAATTTCCCTTCCTTAAG | D39 | DFR |  |
|  | | | |  |
| Construction of IL 127 (D39 *rio03^ATG-GGG^;rio03^GGG-ATG^-kan)* | | | |  |
| ILp356 | atgagaaatatcggacaagc | D39 | UFR |  |
| ILp446 | acggatcctaACCCACTACAGTTGACAAAG | D39 | UFR |  |
| ILp452 | tttaccataataggatccgtttgatttttaatggataatgtgatataatctttaaatactgtagaaaagaggaaggaaataataa | P_c_ | P_c_ | Primer contains P_c_ sequence |
| BRp205 | gaaataataataggatccgtttgatttttaatggataatg | R6 *cbp3*::kan-rpsL^+^ | Kan cassette |  |
| ILp448 | ctttttttcaCTAAAACAATTCATCCAGTAAAATATAATATTTTATTTTC | R6 *cbp3*::kan-rpsL^+^ | Kan cassette |  |
| ILp449 | attgttttagTGAAAAAAAGCTAAGCTCGAGAAAG | D39 | DFR |  |
| ILp202 | GGAGCAATTTCCCTTCCTTAAG | D39 | DFR |  |
|  | | | |  |
| Construction of IL101 (D39 *rio83^ATG-GGG^-spec*) | | | |  |
| ILp170 | tctatacataataagtaaaggaggaatttgtgggatataccatcgtttagaataagtctatatt | D39 | rio83^GGG^ | PCR stitching IL161/IL166 |
| ILp161 | AGTTAAGAGAGGAATCTTCATAAATTATAC | D39 | rio83^GGG^ | PCR stitching IL161/IL166 |
| ILp169 | aatatagacttattctaaacgatggtatatcccacaaattcctcctttacttattatgtataga | D39 | rio83^GGG^ | PCR stitching IL161/IL166 |
| ILp166 | AAGACGTGGTTGTTTTTGAATC | D39 | rio83^GGG^ | PCR stitching IL161/IL166 |
| ILp302 | TAAATTTTTCCTGATTGAAACACC | D39 | UFR |  |
| ILp338 | cagaaaaaATGAAAATTAAGAAATTATTGAAAATGGTTATTC | D39 | UFR |  |
| ILp339 | tcttaattttcatTTTTTCTGTTTCCTTTCATTTTTTATTC | D39 | rio83^GGG^ |  |
| ILp340 | tcccatctcatATTGTTCCTCCTAGAAAAATGTG | D39 | rio83^GGG^ |  |
| ILp341 | ggaacaatATGAGATGGGATTATGGACAAATTTTTAAAG | D39 | Middle fragment |  |
| ILp342 | aaaatcgaTTATAGGTTTGAAAAATCATTAATTTCTTTTTC | D39 | Middle fragment |  |
| ILp343 | tcaaacctataaTCGATTTTCGTTCGTGAATAC | pLZ12spec | Spec |  |
| ILp344 | gagagttattacaaaAAAAAAATTGAAAAAAGTGTTTCCAC | pLZ12spec | Spec |  |
| ILp345 | atttttttTTTGTAATAACTCTCATCATTTTATTATAGTAG |  | DFR | Combined with IL018 |
|  | | | | |
| Construction of IL48 (D39 *rio49-sfGFP)* | | | | |
| ILp171 | ACAGATTTAGCAGCGCGTG | D39 | UFR |  |
| ILp233 | cgaaaatcgaTCATAAAGCAAAAAGTTGGAGC | D39 | UFR |  |
| ILp234 | tgctttatgaTCGATTTTCGTTCGTGAATAC | pLZ12spec | Spec |  |
| ILp235 | tagttcgttcAAAAAAATTGAAAAAAGTGTTTCCAC | pLZ12spec | Spec |  |
| ILp236 | caatttttttGAACGAACTATCTAAACCGAAAC | D39 | sORF |  |
| ILp237 | cacctttgctTAGAGTTACAAAGTACTGATAGGAAAAC | D39 | sORF |  |
| ILp238 | tgtaactctaAGCAAAGGTGAAGAACTGTTTAC | pY71-sfGFP | sfGFP |  |
| ILp239 | aatattgttaTTTTTCGAACTGCGGATG | pY71-sfGFP | sfGFP |  |
| ILp240 | gttcgaaaaaTAACAATATTTTTTAAGGGGGGAC | D39 | DFR |  |
| ILp180 | ACCAATTTATGACTGAGTGC | D39 | DFR |  |
|  | | | | |
| Construction of IL47 (D39 *rio85-sfGFP*) | | | | |
| ILp223 | TGGAATGTCATCCCCATC | D39 | UFR |  |
| ILp224 | caatttttttTTAACATCAAAACAACGTGCC | D39 | UFR |  |
| ILp225 | tttgatgttaaAAAAAAATTGAAAAAAGTGTTTCCAC | pLZ12spec | Spec |  |
| ILp226 | aattatgtcaTCGATTTTCGTTCGTGAATAC | pLZ12spec | Spec |  |
| ILp227 | cgaaaatcgaTGACATAATTTCTTTAAAAGAGTTTCTTTTTATAC | D39 | sORF |  |
| ILp228 | gttcgaaaaaTAGGCTCAAATTTTAGTCATGAAAG | D39 | sORF |  |
| ILp229 | tttgagcctaTTTTTCGAACTGCGGATG | pY71-sfGFP | sfGFP |  |
| ILp230 | tttgagacctAGCAAAGGTGAAGAACTGTTTAC | pY71-sfGFP | sfGFP |  |
| ILp231 | cacctttgctAGGTCTCAAATTTTCCGCAG | D39 | DFR |  |
| ILp232 | ACAGCAGCTTTCAGCCTC | D39 | DFR |  |
|  | | | | |
| Construction of IL61 (D39 *rio106-sfGFP*) | | | | |
| ILp312 | AACACAAGAACAAAAGATACTCC | D39 | UFR |  |
| ILp313 | atttttttACTTCAAGTAAAAAGTTATTCAAGAAAATC | D39 | UFR |  |
| ILp314 | tttttacttgaagtAAAAAAATTGAAAAAAGTGTTTCCAC | pLZ12spec | Spec |  |
| ILp315 | aagctaaaTCGATTTTCGTTCGTGAATAC | pLZ12spec | Spec |  |
| ILp316 | aacgaaaatcgaTTTAGCTTTTGAACTAGTCGTTG | D39 | sORF |  |
| ILp317 | gcagttcgaaaaaTAAAATTCGTAAAAGAATATCATGAATG | D39 | sORF |  |
| ILp318 | gaattttaTTTTTCGAACTGCGGATG | pY71-sfGFP | sfGFP |  |
| ILp319 | atttaagaAGCAAAGGTGAAGAACTGTTTAC | pY71-sfGFP | sfGFP |  |
| ILp320 | ttcacctttgctTCTTAAATTCTGTGCTGTATTTGC | D39 | DFR |  |
| ILp321 | GTGGGGATAATTACTGCAATAATC | D39 | DFR |  |
|  | | | | |
| Construction of IL63 (D39 *rio48-sfGFP*) | | | | |
| ILp282 | ACCCACAGGAAACAGCCG | D39 | UFR |  |
| ILp283 | aaaatcgaTCTTACAAGACTTTTGCTGAAGTG | D39 | UFR |  |
| ILp284 | agtcttgtaagaTCGATTTTCGTTCGTGAATAC | pLZ12spec | Spec |  |
| ILp285 | ttgggcttccctAAAAAAATTGAAAAAAGTGTTTCCAC | pLZ12spec | Spec |  |
| ILp286 | atttttttAGGGAAGCCCAAAGGCTTC | D39 | sORF |  |
| ILp287 | acctttgctAAGAGACCCCCTGAAATAAGC | D39 | sORF |  |
| ILp288 | ggtctcttAGCAAAGGTGAAGAACTGTTTAC | pY71-sfGFP | sfGFP |  |
| ILp289 | cgaggtcaTTTTTCGAACTGCGGATG | pY71-sfGFP | sfGFP |  |
| ILp290 | ttcgaaaaaTGACCTCGAAGGGCTAGAG | D39 | DFR |  |
| ILp291 | CATATACCTTGTGGTAGTCATTATTTG | D39 | DFR |  |
|  | | | | |
| Construction of IL68 (D39 *rio01-sfGFP*) | | | | |
| ILp292 | TGTATGTGAGAAAGGAAGAGC | D39 | UFR |  |
| ILp293 | aaaatcgaAAAATTATTTTTTCCGTCTATTTCTAAAAAAG | D39 | UFR |  |
| ILp294 | gaaaaaataattttTCGATTTTCGTTCGTGAATAC | pLZ12spec | Spec |  |
| ILp295 | ttcattttaaaagcAAAAAAATTGAAAAAAGTGTTTCCAC | pLZ12spec | Spec |  |
| ILp296 | atttttttGCTTTTAAAATGAATAGGAATGTGATATAATAAATAG | D39 | sORF |  |
| ILp297 | tcacctttgctTTCTATCTTATTTATTCGGAAAAAGC | D39 | sORF |  |
| ILp298 | agatagaaAGCAAAGGTGAAGAACTGTTTAC | pY71-sfGFP | sfGFP |  |
| ILp299 | ctagattaTTTTTCGAACTGCGGATG | pY71-sfGFP | sfGFP |  |
| ILp300 | cagttcgaaaaaTAATCTAGAATAAATGATAATAGAAAAGAGAAAATTATG | D39 | DFR |  |
| ILp301 | ACGGTTAAAATCAAGTCCTG | D39 | DFR |  |
|  | | | | |
| Construction of IL59 (D39 *rio83-sfGFP*) | | | | |
| ILp302 | TAAATTTTTCCTGATTGAAACACC | D39 | UFR |  |
| ILp303 | atttttttGTCTATATTTAAAATAGAAGTTATAAGGAATC | D39 | UFR |  |
| ILp304 | ttttaaatatagacAAAAAAATTGAAAAAAGTGTTTCCAC | pLZ12spec | Spec |  |
| ILp305 | agttcgaaaaataaTCGATTTTCGTTCGTGAATAC | pLZ12spec | Spec |  |
| ILp306 | aaaatcgaTTATTTTTCGAACTGCGGATG | pY71-sfGFP | sfGFP |  |
| ILp307 | gtttagaaAGCAAAGGTGAAGAACTGTTTAC | pY71-sfGFP | sfGFP |  |
| ILp308 | tcacctttgctTTCTAAACGATGGTATATCATACAAATTC | D39 | DFR |  |
| ILp309 | TTGTTTCAGTTCTGTTTTTCAATAC | D39 | DFR |  |
|  | | | | |
| Construction of IL64 (D39 *rio97-sfGFP*) | | | | |
| ILp329 | AGCGCCTCTGTAACAAGG | D39 | UFR |  |
| ILp330 | tcacctttgctACAAGTATCGTAGTCCATTGG | D39 | UFR |  |
| ILp331 | atacttgtAGCAAAGGTGAAGAACTGTTTAC | pY71-sfGFP | sfGFP |  |
| ILp306 | aaaatcgaTTATTTTTCGAACTGCGGATG | pY71-sfGFP | sfGFP |  |
| ILp305 | agttcgaaaaataaTCGATTTTCGTTCGTGAATAC | pLZ12spec | Spec |  |
| ILp332 | agctatagtaatttgAAAAAAATTGAAAAAAGTGTTTCCAC | pLZ12spec | Spec |  |
| ILp333 | atttttttCAAATTACTATAGCTGTTTCTACGG | D39 | DFR |  |
| ILp334 | GACAGGGAGGCTTTCTCATC | D39 | DFR |  |
|  | | | | |
| Construction of IL66 (D39 *rio03-sfGFP*) | | | | |
| ILp323 | CCAAGGCTGTCAAAGATG | D39 | UFR |  |
| ILp324 | tcacctttgctGAATAGATAACACCGTTCTAAATTAC | D39 | UFR |  |
| ILp325 | atctattcAGCAAAGGTGAAGAACTGTTTAC | pY71-sfGFP | sfGFP | Combined with IL306 |
| ILp326 | gatctattcctagAAAAAAATTGAAAAAAGTGTTTCCAC | pLZ12spec | Spec | Combined with IL305 |
| ILp327 | atttttttCTAGGAATAGATCATACCAGAGG | D39 | DFR |  |
| ILp328 | CTCAAAACACTGTTTTGAGGTTG | D39 | DFR |  |
|  | | | | |
| Construction of IL45 (D39 *rio56-sfGFP*) | | | | |
| ILp203 | AACTGCCTTAACTGGAGC | D39 | UFR |  |
| ILp204 | caatttttttGATGAAAAAGCTTTGCAATTCATG | D39 | UFR |  |
| ILp205 | ctttttcatcAAAAAAATTGAAAAAAGTGTTTCCAC | pLZ12spec | Spec |  |
| ILp206 | tccaaaaaatTCGATTTTCGTTCGTGAATAC | pLZ12spec | Spec |  |
| ILp207 | cgaaaatcgaATTTTTTGGAAAATAAAAATCCCAGTCG | D39 | sORF |  |
| ILp208 | gttcgaaaaaTAACGCAGGAGCGGACCTTG | D39 | sORF |  |
| ILp209 | tcctgcgttaTTTTTCGAACTGCGGATG | pY71-sfGFP | sfGFP |  |
| ILp210 | aaggaggtcaAGCAAAGGTGAAGAACTGTTTAC | pY71-sfGFP | sfGFP |  |
| ILp211 | cacctttgctTGACCTCCTTATGCTCAATAG | D39 | DFR |  |
| ILp212 | TGCCAGAATTTGTGAAAAAAC | D39 | DFR |  |
|  | | | | |
| Construction of IL46 D39 *rio82-sfGFP* | | | | |
| ILp213 | TTCAACTTGTCTTTTTCTCTGC | D39 | UFR |  |
| ILp214 | caatttttttATCCTTCTTTCTTGCATTGATTAC | D39 | UFR |  |
| ILp215 | aaagaaggatAAAAAAATTGAAAAAAGTGTTTCCAC | pLZ12spec | Spec |  |
| ILp216 | ctttctatttTCGATTTTCGTTCGTGAATAC | pLZ12spec | Spec |  |
| ILp217 | cgaaaatcgaAAATAGAAAGTGAATTTAGATTCTTTACTTTTAG | D39 | sORF |  |
| ILp218 | gttcgaaaaaTAGTATAAACAGAAGAGAGAGCG | D39 | sORF |  |
| ILp219 | gtttatactaTTTTTCGAACTGCGGATG | pY71-sfGFP | sfGFP |  |
| ILp220 | agcaaaaaacAGCAAAGGTGAAGAACTGTTTAC | pY71-sfGFP | sfGFP |  |
| ILp221 | cacctttgctGTTTTTTGCTTCCTTCTTTTGG | D39 | DFR |  |
| ILp222 | ACGGTGAGAAGGCAGTTC | D39 | DFR |  |
|  | | | | |
| Construction of IL81 (D39 wild-type *bgaA::* P_1517_*^rio83^*^-GGG,^ *^rio84^*^-GGG^-*luxAB-P_c_-kan*) | | | | |
| ILp166 | aattcttcaaatattttttaaagcccccgactttttccctcctttttgatataataataccac | D39 | rio84^GGG^ | PCR stitching IL161/IL166 |
| ILp167 | gtggtattattatatcaaaaaggagggaaaaagtcgggggctttaaaaaatatttgaagaatt | D39 | rio84^GGG^ | PCR stitching IL161/IL166 |
| ILp168 | aatatagacttattctaaacgatggtatatcccacaaattcctcctttacttattatgtataga | D39 | rio84^GGG^ | PCR stitching IL161/IL166 |
| ILp161 | AGTTAAGAGAGGAATCTTCATAAATTATAC | D39 | rio84^GGG^ | PCR stitching IL161/IL166 |
| ILp264 | ACAGCAGCTATCGTTCTTG | D39 | 5’ *bga* |  |
| ILp141 | gaggaacaatAAAACCCTCCTTATATTATATTTAGTGC | D39 | 5’ *bga* |  |
| ILp142 | ggagggttttATTGTTCCTCCTAGAAAAATGTGAATATTTTC | PCR stitching reaction of rio84^GGG^/rio83^GGG^ | P_1517_ |  |
| ILp143 | caaacttcatTTTTTCTGTTTCCTTTCATTTTTTATTCAAG | PCR stitching reaction of rio84^GGG^/rio83^GGG^ | P_1517_ |  |
| ILp144 | aacagaaaaaATGAAGTTTGGAAATATTTGTTTTTCG | pJC156 | luxAB |  |
| ILp265 | acggatcctaTTATGGTAAATTCATTTCGATTTTTTG | pJC156 | luxAB |  |
| ILp452 | tttaccataataggatccgtttgatttttaatggataatgtgatataatctttaaatactgtagaaaagaggaaggaaataataa | P_c_ | P_c_ | Primer contains P_c_ sequence |
| BR205 | gaaataataataggatccgtttgatttttaatggataatg | R6 *cbp3*::kan-rpsL^+^ | Kan cassette |  |
| ILp280 | gtaagttcttCTAAAACAATTCATCCAGTAAAATATAATATTTTATTTTC | R6 *cbp3*::kan-rpsL^+^ | Kan cassette |  |
| ILp281 | attgttttagAAGAACTTACTTTCTCATAAACCAG | D39 | 3’ *bga* |  |
| ILp269 | GAACTTCTACCACTTCACGG | D39 | 3’ *bga* |  |
|  | | | | |
| Construction of IL93 (D39 *bgaA::* P_1517_*^rio83^*^-GGG,^ *^rio84^*^-GGG^ -*luxAB-P_c_-kan-P_c_-rio84*) | | | | |
| ILp387 | acggatcctaCTAAAACAATTCATCCAGTAAAATATAATATTTTATTTTC | Strain IL81 | *3’ bga-P_1517_-luxAB* | Combined with IL264 |
| ILp388 | attgttttagTAGGATCCGTTTGATTTTTAATGG | Strain IL81 | P_c_ |  |
| ILp389 | taaagcccatTTATTATTTCCTTCCTCTTTTCTACAG | Strain IL81 | P_c_ |  |
| ILp390 | gaaataataaATGGGCTTTAAAAAATATTTGAAGAATTTAC | D39 | *rio84* |  |
| ILp391 | gtaagttcttTTATCCCCAAAACCATGTTTC | D39 | *rio84* |  |
| ILp392 | ttggggataaAAGAACTTACTTTCTCATAAACCAG | D39 | 3’ *bga* | Combined with IL269 |
|  | | | | |
| Primers for qRT-PCR | | | | |
| ILp121 | tgagcggcagcactatctcc |  | *gyrA* | Housekeeping gene |
| ILp122 | tgggtaaatatcacccacacggg |  | *gyrA* | Housekeeping gene |
| ILp402 | GCTTGCCTAGGGTGACAGTA |  | *srf-02* | Noncoding RNA srf-02 |
| ILp403 | ACAAGAGTCCTGCCCACTAA |  | *srf-02* | Noncoding RNA srf-02 |
| ILp151 | CCGTTGCCCAATTACCATGAG |  | *spv_1517* |  |
| ILp152 | GGAGAAGCTCGTAGTGGAGA |  | *spv_1517* |  |
|  | | | | |
| Primers for 5’-RACE | | | | |
| BRp311 | GCTAATCATTGCAAGCAGTGGTATCAACGCAGAGTACATGGG |  |  | Template Switching Oligonucleotide (TSO) |
| BRp312 | CATTGCAAGCAGTGGTATCAAC |  |  | TSO Primer |
