# Supplementary material for: Discovery of Unannotated Small Open Reading Frames in Streptococcus pneumoniae D39 Involved in Quorum Sensing and Virulence Using Ribosome Profiling
Source: mBio. 2022 Jul 19;13(4):e01247-22. doi: 10.1128/mbio.01247-22 (PMC9426450; doi:10.1128/mbio.01247-22)
Supplement: Supplementary file 3 [file mbio.01247-22-s0008.docx]

RIO116	1085207-1085290	spv_1059, spv_1060	-	2.5	ATG	ATGGTCTGCTTTTTTAGTTTTTATACTCTTCGAAAATCTCTTCAAACCACGTCAGCGTCGCCTTACTGTATGTATGGTTACTGA	MVCFFSFYTLRKSLQTTSASPYCMYGY	27	3.1
RIO117	1250001-1250069	spv_1217, spv_1218	-	13	ATG	ATGAAGGTGGTACCGCGGTTTTTCGCCCTTCGTGATATGAGCTTGTCTTTTGATTTTTGGAGGTGTTGA	MKVVPRFFALRDMSLSFDFWRC	23	2.7
RIO118	1814026-1814076	spv_1828, spv_1829	-	21	ATG	ATGAAAACAGAACTGTTTCTTTTGCTATTAGTTCAAAAGGAGAAAAAAATGA	MKTELFLLLLVQKEKK	16	1.9
RIO119	150808-150888	spv_0144, spv_0145	-	1338	ATG	ATGAAGAAAAGAAAAATCCAACTGATACTCCTACTTATTTCGGAGTGGGTTATTGTCATTCCATTTTTAACTAATCTATAA	MKKRKIQLILLLISEWVIVIPFLTNL	27	3.1
RIO120	150730-150771	spv_0144, spv_0145	-	47	ATG	ATGTTAATTGTGGATTTTCTAATACCTGCAGAGATTGGATAA	MLIVDFLIPAEIG	13	1.4
RIO121	150391-150477	spv_0144, spv_0145	-	16.7	TTG	TTGATAAAAAAATCAGATCTTGATTGCTCAAGAAGGGCATGGTATTGCAAAAGAGATAGAGTTCAAACCAGGTCCAAGACTCGATAG	MIKKSDLDCSRRAWYCKRDRVQTRSKTR	28	3.4
RIO122	149626-149673	spv_0143, spv_0144	-	0.25	ATG	ATGGAGCGGTTACAAAGAACCTACTTTCTATTAAACAGTATACTATGA	MERLQRTYFLLNSIL	15	1.8
